# A double-labeling marker-based method for estimating inbreeding and parental genomic components in a population under conservation

**DOI:** 10.5713/ajas.19.0035

**Published:** 2019-07-01

**Authors:** Wenting Li, Mengmeng Zhang, Kejun Wang, Yunfeng Lu, Hui Tang, Keliang Wu

**Affiliations:** 1 College of Animal Science and Technology, China Agricultural University, Beijing, 100193, China; 2 College of Animal Sciences and Veterinary Medicine, Henan Agricultural University, Zhengzhou, 450002, China; 3 School of Life Science and Technology, Nanyang Normal University, Nanyang Henan, 473061, China; 4 College of Animal Science and Veterinary Medicine, Shandong Agricultural University, Tai’an, 271018, China

**Keywords:** Genetic Diversity, Double-labelling Method, Conservation Programs, Parental Genomic Components

## Abstract

**Objective:**

The objective of a conservation program is to maintain maximum genetic diversity and preserve the viability of a breed. However, the efficiency of a program is influenced by the ability to accurately measure and predict genetic diversity.

**Methods:**

To examine this question, we conducted a simulation in which common measures (i.e. heterozygosity) and novel measures (identity-by-descent probabilities and parental genomic components) were used to estimate genetic diversity within a conserved population using double-labeled single nucleotide polymorphism markers.

**Results:**

The results showed that the accuracy and sensitivity of identity-by-state probabilities and heterozygosity were close to identity by descent (IBD) probabilities, which reflect the true genetic diversity. Expected heterozygosity most closely aligned with IBD. All common measures suggested that practices used in the current Chinese pig conservation program result in a ~5% loss in genetic diversity every 10 generations. Parental genomic components were also analyzed to monitor real-time changes in genomic components for each male and female ancestor. The analysis showed that ~7.5% of male families and ~30% of female families were lost every 5 generations. After 50 generations of simulated conservation, 4 male families lost ~50% of their initial genomic components, and the genomic components for 24.8% of the female families were lost entirely.

**Conclusion:**

In summary, compared with the true genetic diversity value obtained using double-labeled markers, expected heterozygosity appears to be the optimal indicator. Parental genomic components analysis provides a more detailed picture of genetic diversity and can be used to guide conservation management practices.

## INTRODUCTION

The primary goal of a conservation program is to maintain the maximum genetic diversity of a conserved stock [1], effectively insuring the population against changes in the environment or shifts in market preferences [2]. Stock conservation has typically involved limited numbers of breeding animals and lines. Loss of genetic diversity in these populations can occur due to inbreeding, genetic drift, selection, or other factors [3,4]. It has therefore been necessary to develop reliable methods to measure and detect changes in genetic diversity in order to safeguard conserved stocks and guide conservation programs.

Several approaches, including pedigree-based and molecular marker-based methods, have been applied to estimate genetic diversity [2,5]. However, the pedigree-based method has unavoidable deficiencies. Co-ancestry coefficients based on pedigree information are assumed to be constants, such as full-sib (0.5) and half-sib (0.25), without considering Mendelian sampling [5,6]. Pedigree information is also commonly inaccurate or incomplete. In contrast, the marker-based method makes use of a large number of markers to estimate the co-ancestry coefficient, providing a better estimate of genetic diversity. The accuracy of the estimate depends on the density of markers, and is low when small numbers of markers are used [5]. Early studies with microsatellite markers suggested that marker-based methods were inferior to pedigree-based methods for maintaining genetic diversity [7,8]. However, more recent research shows that marker-based methods using whole-genome single nucleotide polymorphisms (SNPs) can maintain diversity better than pedigree-based methods if marker density is high enough [9,10]. Further study has confirmed that an SNP density of 500 SNPs/Morgan is sufficient [11]. A marker-based method is therefore preferable for estimating genetic diversity for conservation, given appropriate marker density.

Due to the fact that SNPs are bi-allelic, optimal measures with sufficient sensitivity and accuracy are essential for monitoring the genetic diversity in conserved stocks. Measures based on gene frequency, such as the observed and expected heterozygosity (*Ho* and *He*), observed and effective number of alleles (*Ao* and *Ae*), and polymorphism information content (*PIC*), have been used for this purpose [12–14]. Another class of measures, based on the probability of alleles being identical between individuals, has also been applied [5]. These include identity by descent (IBD) probability and identity by state (IBS) probability. If two alleles drawn randomly from two individuals are IBD, this indicates that the alleles have descended from a common ancestor [4]. Measures based on IBD perform better than those based on gene frequency in situations with either high or low marker density [5]. In fact, the nature of inbreeding estimates derived from pedigrees are also based on IBD probabilities. However, the pedigree-based method relies on a base population, while IBD and IBS probabilities can be directly estimated with whole-genome SNPs without reference to the base population [4].

Large numbers of SNPs are now available in commercial pig breeds to estimate effective population size and genetic diversity [15]. However, diversity has been estimated based on different measures and expressed using different scales, without a golden standard for comparison. In order to maximize the effectiveness of diversity conservation efforts that are being applied to conserve Chinese pig breeds, we established a simulated conserved population and genotyped it using double-labeled markers (one label for allele information and the other for family information). Furthermore, we identified the optimized measure for estimating genetic diversity, and monitored dynamic changes of diversity using parental genomic components. The results provide insights into current efforts to conserve Chinese pig stocks, and might be used to guide and improve conservation strategies.

## MATERIALS AND METHODS

A base population at mutation-drift equilibrium was prepared using *in silico* simulation. The base population was then managed for 50 generations. Genetic diversity parameters, such as heterozygosity, IBD probabilities, IBS probabilities, and genealogical coefficients (F), were measured to assess the current practices used in the Chinese pig conservation program (see methods as below). All simulation data was generated using Fortran 90 codes, with averaging over 100 replicates.

### The single nucleotide polymorphism architecture of the initial conserved stock

The base population was generated by simulation through 5,000 generations of random mating until reaching mutation-drift equilibrium. This population is defined as generation zero (*t* = 0). The population comprised 120 individuals, consisting of 20 males and 100 females. Population size was kept constant across generations. We simulated 1,200 SNPs per chromosome and these SNPs were evenly distributed across each of the 18 chromosome in the pig genome. All marker loci were initially fixed at the “1” allele (*t* = −5,000) and selected loci were permitted to mutate to allele “2”. The mutation rate per locus in each generation was *μ* = 2.5×10^−4^. The number of new mutations in each generation was drawn from a Poisson distribution with mean 2*N_e_n_c_μn_l_*, where *N_e_* was the effective population size, *n_c_* was the total number of chromosomes, and *n_l_* was the marker number per chromosome [11]. Mutations occurred randomly across markers, chromosomes, and individuals. Mutated alleles were allowed to return to their previous state, but reversion occurred very rarely. When producing gametes with recombination, the number of crossovers in each chromosome was determined using a Poisson distribution with a mean of 1. After 5,000 generations, markers with a minor allele frequency >0.05 and call rate >90% were identified, yielding 1,000 markers per chromosome. These were used for further analysis. We also confirmed that the population reached mutation-drift equilibrium by monitoring the genetic diversity parameters of the population over the 5,000 generations. Diversity measurements were relatively stable upon reaching *t* = 0 [11].

### Simulated management of a conserved stock

The initial population was managed for 50 generations. The mutation rate during the conservation period was set to 2.5× 10^−6^. The management strategy was identical to the one currently in use by the swine conservation program in China. Briefly, the strategy is as follows. The conserved population consists of 12 males from non-related families and more than 100 females. Candidates for each generation are randomly picked using the “equal procedure”. That is, every male family retains one boar, and randomly retains gilts from different sows (R:F). The population size is kept constant during the conservation period. *He*, *Ho*, *Ao*, *Ae*, *PIC*, and number of rare alleles (RA) were used to measure the diversity over the whole genome and for each chromosome, which were calculated as previous study [12].

In addition, the rate of decline for each diversity parameter was calculated between adjacent generations. To express the rate of decline, we determined the number of generations required to reduce a given parameter by 5% from its initial value at *t* = 0. To observe the dynamic changes of SNP distribution, SNP frequencies were sorted into 10 bins. Genealogical inbreeding coefficients were calculated using the formula as follows with the assumption that the individuals were unrelated at *t* = 0.

$$\begin{array}{l} \text{F}=1-{\left(1-\Delta \text{F}\right)}^{t}\\ \Delta \text{F}=1/8{\text{N}}_{\text{m}}+1/8{\text{N}}_{\text{f}} \end{array}$$

Of which, *t*, generation; N_m_, male number of conservation population; N_f_, female number of conservation population.

### Identity by descent probabilities

The IBD probabilities were first introduced as estimates of genetic diversity based on neutral SNP markers [5]. Genetic diversity in Engelsma’s study was estimated using IBD probabilities between haplotypes that were reconstructed from the genotypes [16]. Here, we developed a new method to estimate true IBD probabilities in a simulated population by defining IBD to mean that a DNA marker i) showed identical sequences in two or more individuals, and also ii) originated from the same ancestor. Because we distinguished the male and female parental origins for each marker throughout the simulation, all markers were double-labeled so that we could not only observe the allele status of these markers but also obtain the parental origin for each. The IBD loci were determined based on allele status and parental labels. If markers from different individuals had the same allele status as well as the same origin, we treated them as IBD loci. In contrast, if either the allele status or origin were not the same, the loci would be discarded for the purpose of evaluating IBD probabilities. The IBD probabilities were calculated as G-IBD/(G-hom + G-her), where G-IBD was the number of IBD loci for the whole genome, and G-hom and G-her represented the number of homozygotes and heterozygotes in the whole genome, respectively. However, IBS loci were determined only by allele status. The formula for IBS probabilities was ([G-IBS] – [G-IBS]*_t_*_=0_)/(G-hom + G-her), where G-IBS was the number of IBS loci in the whole genome, and (G-IBS)*_t_*_=0_ was the number of IBS loci in the whole genome at *t* = 0. Similarly, IBD and IBS probabilities were calculated for each chromosome.

### Estimation of kinship and effective population size

Simulated SNP information of conserved stock were exported after managing for 50 generations. The kinship between individuals were calculated with GCTA v1.92.1beta6 [17] and reshaped into genomic relationship matrix. Meanwhile, effective population size (*Ne*) for each 5 generations were estimated according to the random mating model of linkage disequilibrium using N_E_ESTIMATOR v2.01 [18]. While the theoretical value was also calculated with *Ne* = *N*/(1+F), *N* = 4N_m_N_f_/(N_m_+N_f_). F has been estimated in the above; N_m_, male number of conservation population; N_f_, female number of conservation population.

### Parental genomic components analysis

Using double-labeled markers, we could trace marker origin in all descendants. The proportion of parental genome in the genome of a descendant genome was defined as the parental genomic component (PGC). Using the first male family as an example, PGC was calculated as M1_(_*_t_*_)_/(N_m_×*n_l_*×*n_c_*), where M1 was the number of males with ID = 1 at generation *t*, N_m_ was the population size of males, *n_c_* was the total number of chromosomes, and *n_l_* was the number of markers per chromosome. Meanwhile, all male and female family was calculated as the formula described. The relative genomic components (RGC) for each generation relative to the initial generation was calculated as (PGC_(_*_t_*_=_*_n_*_)_ – PGC_(_*_t_*_=0)_)/PGC_(_*_t_*_=0)_.

## RESULTS

### The dynamics of genomic diversity under conservation

**Figure 1:**
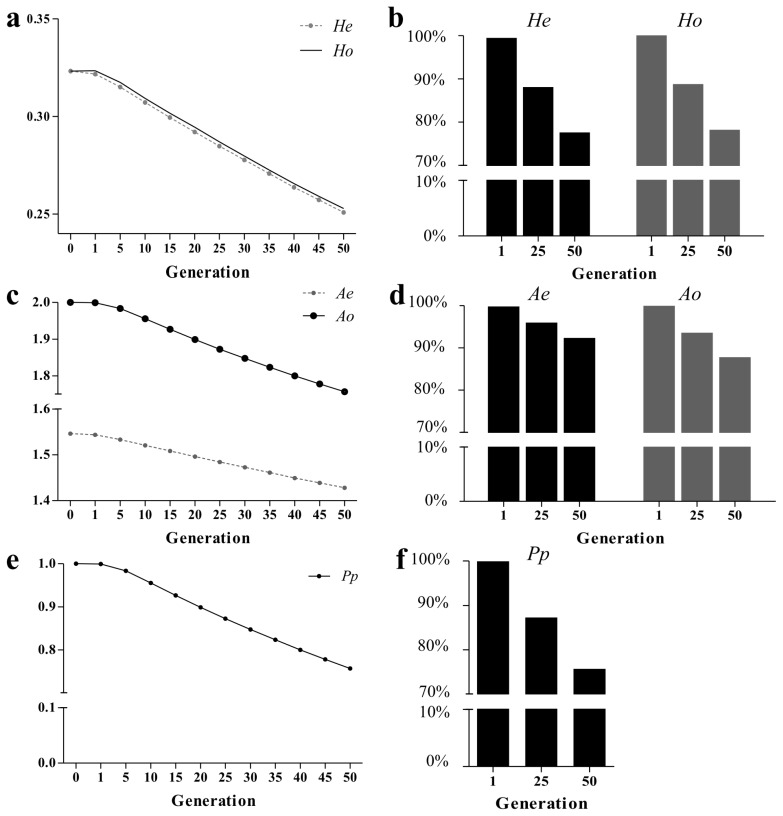
Dynamic changes in genetic diversity across conserved generations. (a–b) Expected (*He*) and observed (*Ho*) heterozygosity. (c–d) Effective allele number (*Ae*) and observed allele number (*Ao*). (e–f) Proportion of polymorphic alleles (*Pp*).

The *He* and *Ho* decreased in the conserved population throughout the 50-generation simulation (Figure 1a–b). The *He* declined from 0.323 at *t* = 0 to 0.251 at *t* = 50, a ~22.4% decline relative to the initial value. Similarly, *Ho* declined by ~21.8%. Genetic diversity was also assessed using measures based on allele number (*Ao*, *Ae*, and *Pp*). The *Ao* decreased from 2 to 1.76 during the simulation, while *Ae* decreased from 1.55 to 1.43, representing reductions of 12.2% and 7.63% from the values at *t* = 0, respectively (Figure 1c–d). The *Pp* fell from the initial value of 1 to 0.757 over the same period (Figure 1e–f), indicating that diversity was lost for ~24.3% of alleles. Table 1 shows the loss in diversity expressed as a series of 5% decreases. For example, 5% of diversity, as measured using *He*, *Ho*, *Ao*, *Ae*, and *Pp*, was lost by the time the simulation reached generations 11, 12, 20, 32, and 11, respectively. The population contained 159 RA (allele frequency <0.05) at *t* = 0. The number of RA increased sharply to 660.52 at *t* = 9, and then fluctuated over a small range (data not shown). Additional genetic diversity parameters for each chromosome were calculated (Supplementary Figure S1), and all chromosomes exhibited dynamic changes similar to those observed for the entire genome.

### Evaluating inbreeding using IBD-based probabilities, IBS-based probabilities, and genealogical coefficients (F)

**Figure 2:**
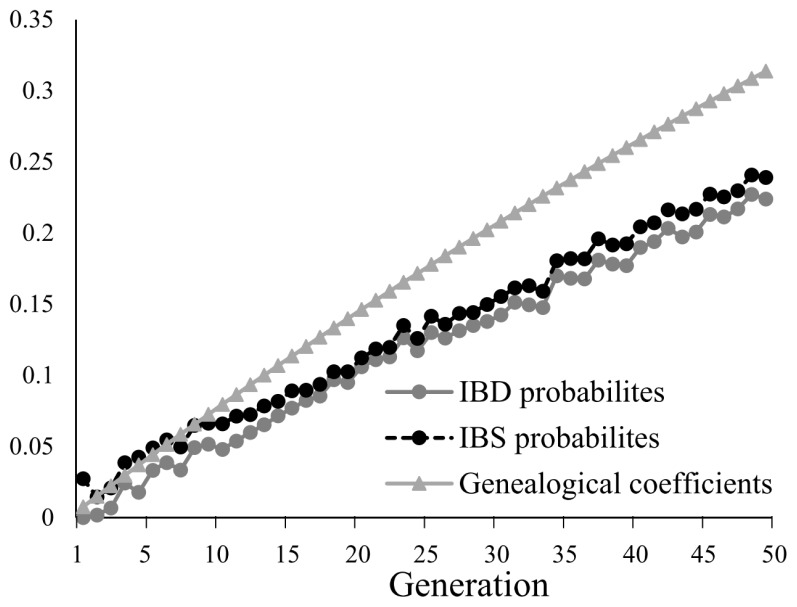
Dynamic changes in IBD probabilities, IBS probabilities, and genealogical coefficients during conservation. IBD, identity by descent; IBS, identity by state.

**Figure 3:**
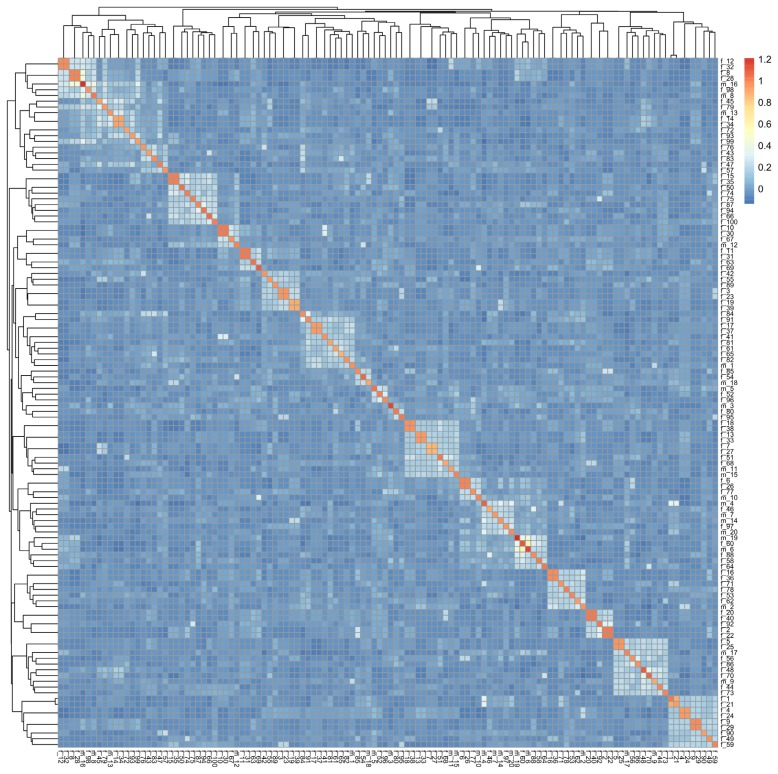
Genomic relationship matrix for individuals from conserved population at the 50th generation.

At *t* = 0, both IBD probabilities and F had values of 0, based on the assumption that the individuals from the base population had no genetic relationship. The results showed that IBD probabilities increased from 0 at *t* = 0 to 0.224 as the population was managed for 50 generations. IBS probabilities were slightly higher than IBD probabilities, ranging from 0.0209 to 0.234. IBS and IBD probabilities had similar dynamics (Figure 2), although the IBS probabilities were always 5% to 10% higher than the IBD probabilities. F increased linearly throughout the conservation period and had the highest values amongst the three coefficients. From the 10th generation, F exceeded both the IBD and IBS probabilities, ultimately reaching 0.314 by the 50th generation. IBD and IBS both increased by about 5% per 11 generations, while F increased at a slower rate, at 5% per 7.83 generations, on average (Table 1). IBD and IBS probabilities were also calculated for individual chromosomes, and fluctuated from one generation to the next (Supplementary Figure S2). Genomic relationship matrix of individuals at *t* = 50 was shown in Figure 3. The kinship ranged from 0 to 1.21. Most individuals showed relative low genomic relationship.

### Fluctuation in allele frequencies and effective population size during conservation

**Figure 4:**
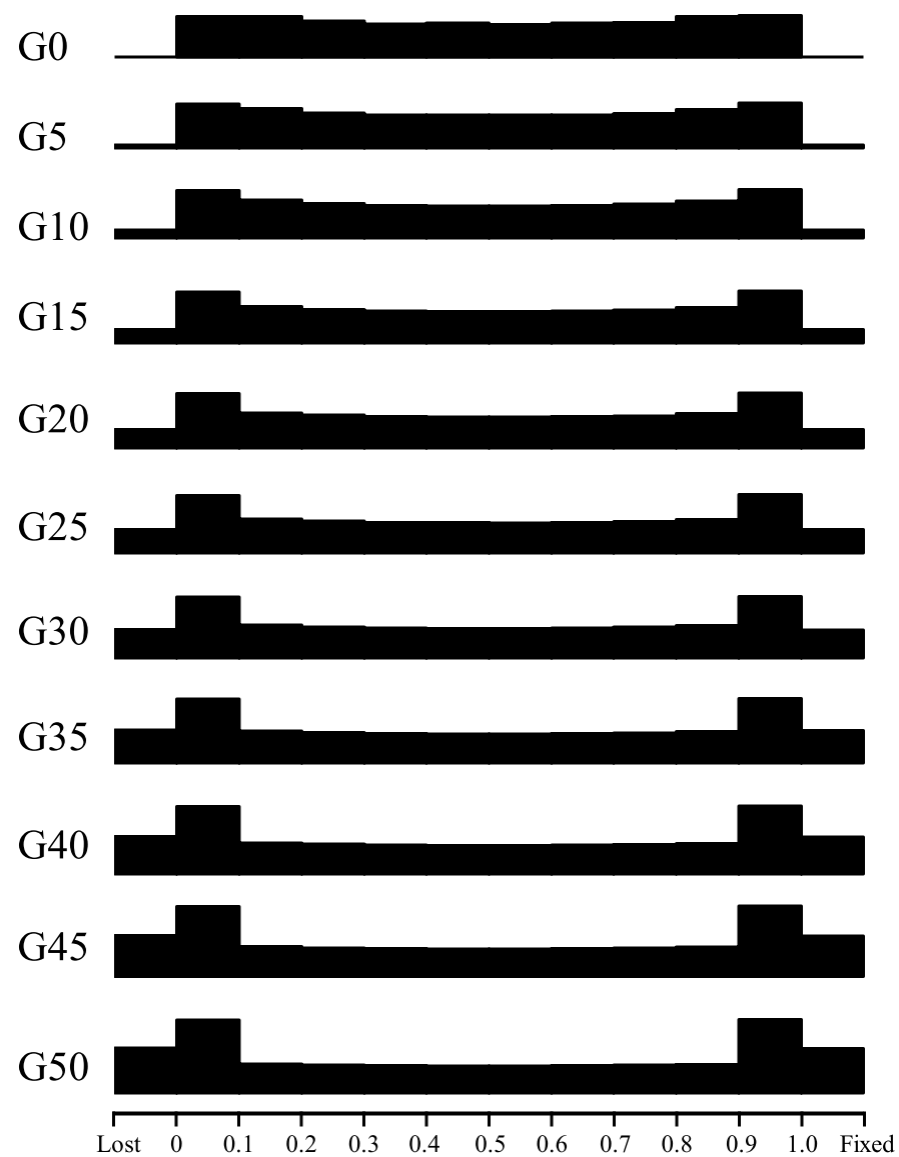
Genomic distribution of allele frequencies every 5 generations. Gene frequencies were sorted into 10 bins. The leftmost bin contains “lost” alleles and the rightmost bin contains “fixed” alleles.

To make it easier to visualize shifts in allele frequencies, we binned the frequency values (from 0 to 1.0) into 10 bins every five generations, with two additional bins to account for “lost” and “fixed” alleles (Figure 4). The distribution of allele frequencies across the genome at *t* = 0 was slightly U-shaped. The SNP differentiation clearly occurs as the management simulation proceeds. By the 5th generation, ~0.81% alleles were fixed or lost. By the 50th generation, alleles with frequencies of 0 to 0.1 and 0.9 to 1.0 were considerably more abundant than those in any other frequency range. Moreover, the numbers of lost and fixed alleles both exceeded 2000.

**Figure 5:**
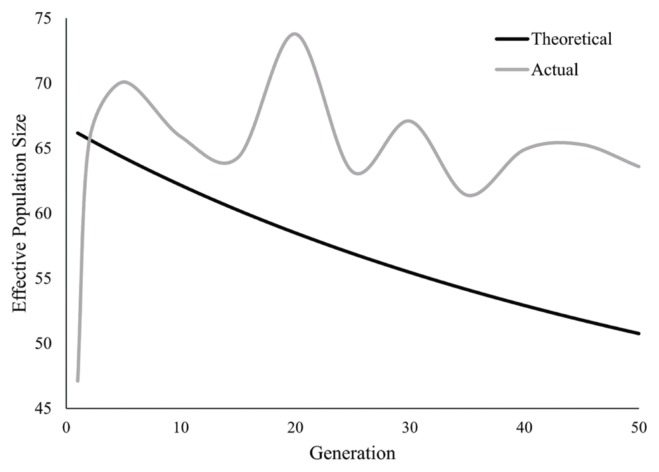
Temporal changes of both theoretical and actual effective population size during conservation. Grey line, theoretical value; Black line, actual value.

Actual effective population size was estimated based on the SNPs of conserved stock for every five generations (Figure 5), which presented as fluctuation across the whole conservation period. The lowest *Ne* was 47.1 at *t* = 1 and the highest was 70.1 at *t* = 5. While the theoretical effective population size was calculated as the formula described in the material section, which showed a continuous and slow decline across the conserved generations.

### Analysis of parental genomic components under conservation management

**Figure 6:**
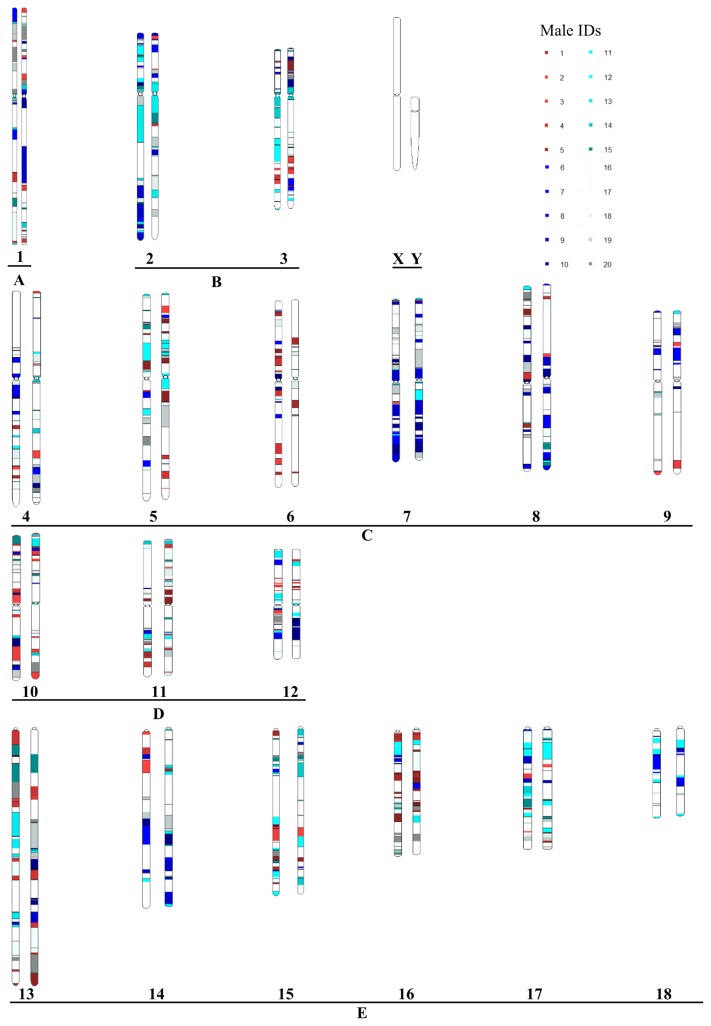
Chromosome ideogram showing parental genomic components. Chromosome IDs are indicated under each chromosome pair. The 20 male families are represented using different colors. Female families are shown in white.

**Figure 7:**
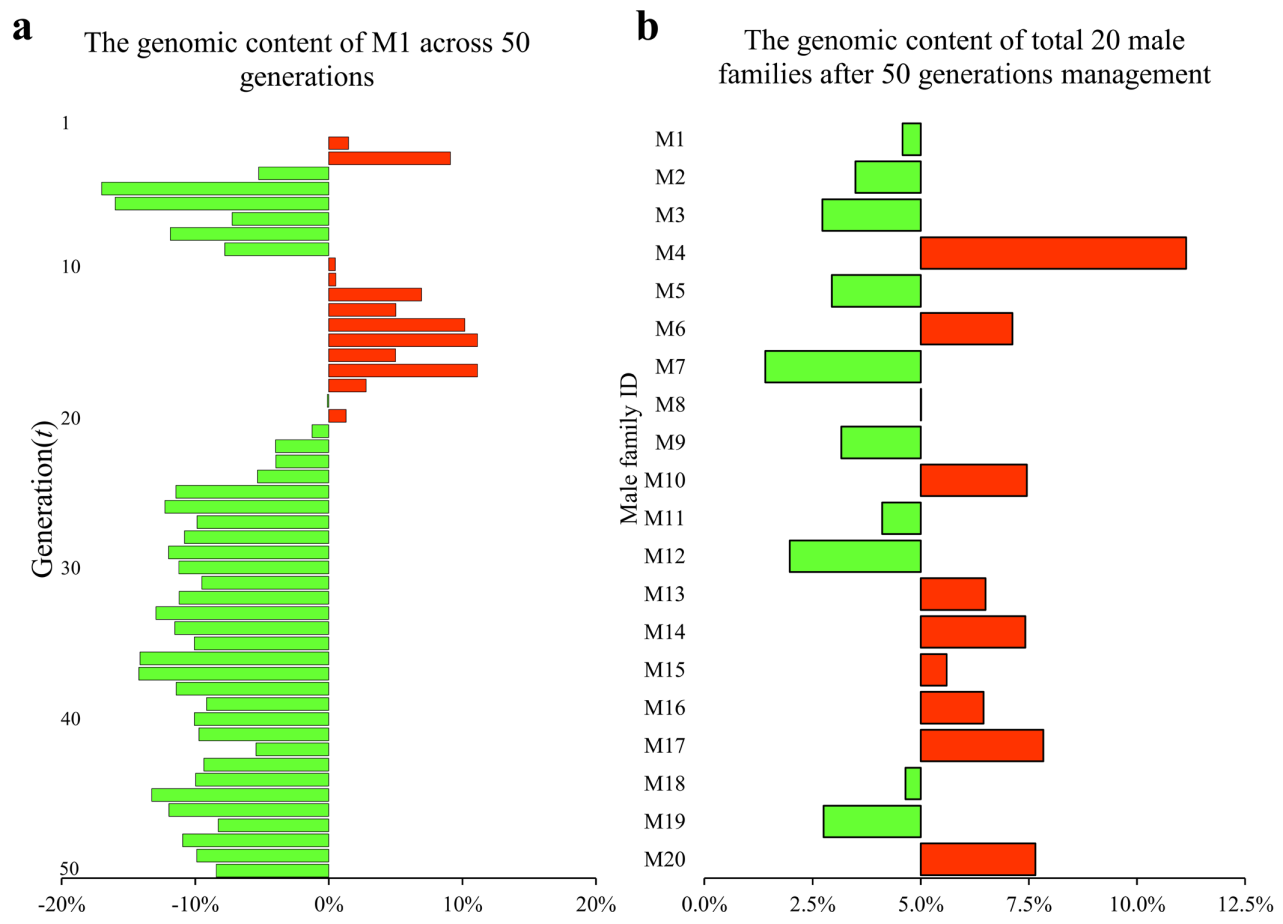
Genomic component contributions of male families. (a) The genomic component contributed by the 1st male family (M1) across 50 generations. (b) The genomic components contributed by all 20 male families at the 50th generation.

To examine the effects of the conservation program on parental genetic components, we tracked parental contributions to the genome throughout the 50-generation conservation period. The proportional contribution for each male was easily obtained using the parental genome tags that accompanied all SNP markers. The genomic components for all male families at the initial generation were set as equal, such that each male family represented 5% of the total male genomic components. The PGC for a randomly selected individual at generation 50 is shown in Figure 6 as a chromosome ideogram. The colors in this figure identify contributions made by each of the 20 founder males, and the white regions represent female family contributions. Male genomic components fluctuated during the conservation period. For example, the genomic component contributed by the 1st male family (M1) was 5.46% at *t* = 3, an increase of ~10% relative to *t* = 0. However, the genomic components derived from M1 at *t* = 5 were 4.15%, a 17% reduction compared to the initial generation (Figure 7a). The genomic component contributions are shown at each generation for each male family in Supplementary Table S1. Overall, after 50 generations, 10 male families increased their genomic components while the others showed a reduction compared to the initial generation. Male family M4 had the maximum contribution (11.1%), about 10-fold higher than M7, which had the minimum contribution of 1.41% (Figure 7b).

**Figure 8:**
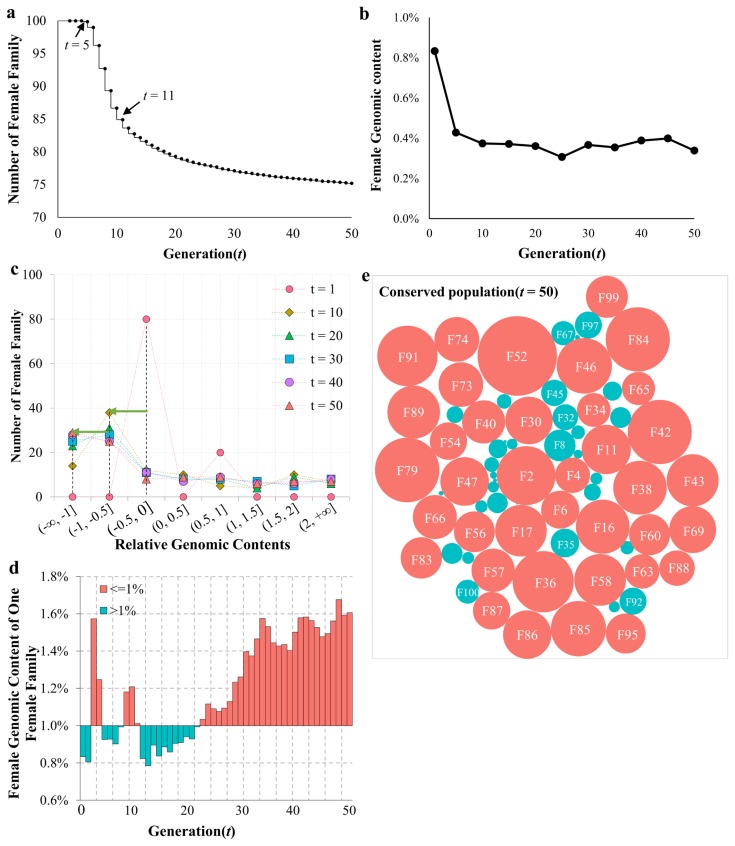
Genomic component contributions of female families. (a) Number of female families during the conservation period. (b) Median of genomic component contributions from 100 female families. (c) The distribution of relative genomic components (RGC) contributed by female families every 10 generations. Different colors represent different generations. The green arrow indicates peak shifts between generations. (d) The genomic component contributed by the 87th female family (F87) across 50 generations. (e) The genomic component contributions of 100 female families at the 50th generation. Red circles indicate genomic components that are higher than the initial values, while blue circles indicate components that are lower.

The conserved stock had 100 female families in total, and the genomic components contributed by each female family was therefore 1% at *t* = 0. We found that the first female family was lost at *t* = 5. The total number of female families decreased from 100 to 75.2 at *t* = 50 (Figure 8a). The rate of decrease for female families was highest from *t* = 6 to *t* = 11, an interval during which 2 to 3 female families were lost per generation. The median value of female genomic components sharply decreased in the first 10 generations, indicating that the genomic components from most female families declined during this stage (Figure 8b). To further examine the female genomic components, RGC values were determined between each generation and the initial generation as described in Methods. The results were sorted into 8 bins, and the numbers of female families in each bin were counted. As shown in Figure 8c, the maximum number of families was located in bin (−0.5, 0] at *t* = 1. Consistently, the peaks moved leftward with increasing generation time. In generations *t* = 10, 20, and 30, the peak had shifted to the adjacent bin, (−1, −0.5]. Finally, at *t* = 40 and 50, the peak moved to bin (−∞, −1]. The genomic components of a representative female family (F87) under conservation are shown in Figure 8d, and hovered around 1%. They declined slightly over the first 17 generations and increased thereafter, relative to the initial generation. Similar fluctuations were observed in other female families (data not shown). Figure 8e shows the relative contributions made by genomic components for all female families that persisted throughout all 50 generations. There were 29 female families being lost during the conservation period, leaving 71 female families in the population. Of these, the genomic components for 38 female families were higher than the initial value, and were lower for the other 33 families.

## DISCUSSION

Simulations have been used to predict the efficiency of strategies that rely on SNP markers to maintain genetic diversity in conserved stock [9,19]. The impact of various factors (such as effective population size and marker density), and the predictors used to estimate genetic diversity (heterozygosity and IBD probabilities), have been discussed in detail [5,11,20]. In this study, we examined PGC and true IBD probabilities using double-labeled SNP markers, and also observed the changes in widely employed parameters for genetic diversity (*He*, *Ho*, *Ae*, *Ao*, and *Pp*) and coalescent genetic diversity (G-IBD, G-IBS, and F) across 50 generations during a simulated conservation program.

Genetic diversity, as estimated using IBS probabilities and heterozygosity, correlated positively with IBD-based genetic diversity (GD_IBD), which is considered to reflect true genetic diversity (Supplementary Figure S3 a–c). Although the IBD probabilities in the initial generation were 0, IBS was not 0. This explains why the IBS-based genetic diversity (GD_IBS) calculated in this study was slightly lower than GD_IBD (Figure 2). A previous study showed that the performance of these predictors is influenced by SNP marker density when they are used to maintain genetic diversity [5]. The marker density in our study was 1,000 SNP/chromosome, a density that is appropriate for maintaining genetic diversity [11], and in practice could be used without imposing high costs for SNP chips. However, the predictors based on allele richness did not perform with adequate sensitivity. Although the correlation between *Ao*, *Ae*, and GD_IBD was strong (Supplementary Figure S3 d–e), the obvious limitation is that these predictors were only able to detect a 5% loss of genetic diversity at the 20th generation (Table 1). Since SNPs are bi-allelic markers, it is possible that allele number was not suitable in this test case. Our recommendation is that GD_IBS and heterozygosity should be used when marker density is on the order of 1,000 SNPs/chromosome. This guideline is consistent with conclusions made by an earlier study [11].

In our study, genetic diversity was estimated using marker loci. Genetic diversity has previously been studied in a simulated neutral genome without selection, or with selectively neutral markers [11,12,20–22]. The Food and Agriculture Organization of the United Nations (FAO) has proposed a subset of 30 microsatellite markers as a standard for monitoring genetic diversity [23,24]. However, the inevitable disadvantage of neutral markers is that deleterious mutations are ignored. The accumulation of deleterious mutations influences breed viability [19,25]. Hall et al [26] confirmed that genetic diversity measurements based on neutral variations are not always sufficient. Methods for predicting genetic diversity could be improved by taking into account both the accumulation of deleterious mutations and the neutralization of markers.

In our simulation, parental genomic components could be tracked using the parental labels accompanying each SNP. In practice, such labels are unavailable. Nevertheless, the parental genomic components estimated in this study are still useful for predicting the loss of genetic diversity in the current Chinese conservation program. Because the population size and management strategy in the simulation are identical to those used in the program, the PGC dynamics we observed potentially reflect actual trends in conserved swine stock. Of most concern is the loss in genetic diversity. For example, the genomic components of three male families (M7, M12, and M19) were reduced by more than 50% at *t* = 5 (Supplementary Table S1). The genomic components of 23% of female families were completely lost (Figure 8a), and 35 female families lost more than 90% of their components for *t* = 5 (data not shown). Moreover, loss of genetic diversity could be observed in other genetic diversity parameters (*He*, *Ho*, *Ae*, *Ao*, and *Pp*), coalescent genetic diversity (G-IBD, G-IBS, and F), and allele frequency.

The simulated conserved stock with its small effective population size had already lost ~10% of genetic diversity at *t* = 5 due to genetic drift. The most direct strategy to rescue the loss of genetic diversity is to enlarge the population size with additional genetic resources from same breed. Accordingly, we could infer that it is appropriate to introduce ~10% additional genetic resources into a managed stock every 5 generations (about 12.5 years in Chinese pig conservation program). This practice would ensure that the conserved stock maintains a high level of genetic diversity in the long term. Additional genetic resources could be obtained from live pigs from another conserved stock of the same breed, or even frozen semen and embryos, thus combining *in vivo* and *in vitro* conservation and reducing overall conservation costs.

We are the first to exploit true IBD probabilities using double-labeled SNP markers in a simulated conservation program. After comparing the performance of GD_IBD and other predictors, we recommend GD_IBS and heterozygosity rather than allele number to monitor dynamic changes in genetic diversity when the marker density is on the order of 1,000 SNPs per chromosome. Additionally, we have incorporated parental genomic components into our assessment of a conservation program, making it possible to develop improved strategies for maintaining conserved stock at a high level of genetic diversity over the long term. Our results also provide a theoretical foundation for combining *in vivo* and *in vitro* conservation to maintain of genetic diversity.

## Supplementary Data











## Figures and Tables

**Table 1:** Generation intervals over which genetic diversity, estimated using various parameters, declined by 5%

Items	IBD	IBS	F	*Ho*	*He*	*Ao*	*Ae*	*Pp*
5%	10	8	7	12	11	20	32	11
10%	21	19	14	22	21	40	66	20
15%	32	30	22	34	32	61	99	30
20%	44	41	30	46	44	81	133	40
25%	54	53	38	54	55	101	166	51
30%	66	64	47	65	67	121	199	61

Left column represented the declined proportion of various parameters relative to initial generation.

IBD, identity by descent; IBS, identity by state; F, genealogical coefficients; *Ho*, observed heterozygosity; *He*, expected heterozygosity; *Ao*, observed number of alleles; *Ae*, effective number of alleles; *Pp*, proportion of polymorphic alleles.
