# Supplementary figures and images for: A double-labeling marker-based method for estimating inbreeding and parental genomic components in a population under conservation
Source: Asian-Australas J Anim Sci. 2019 Jul 1;33(1):12–23. doi: 10.5713/ajas.19.0035 (PMC6946976; doi:10.5713/ajas.19.0035)

Figure S1

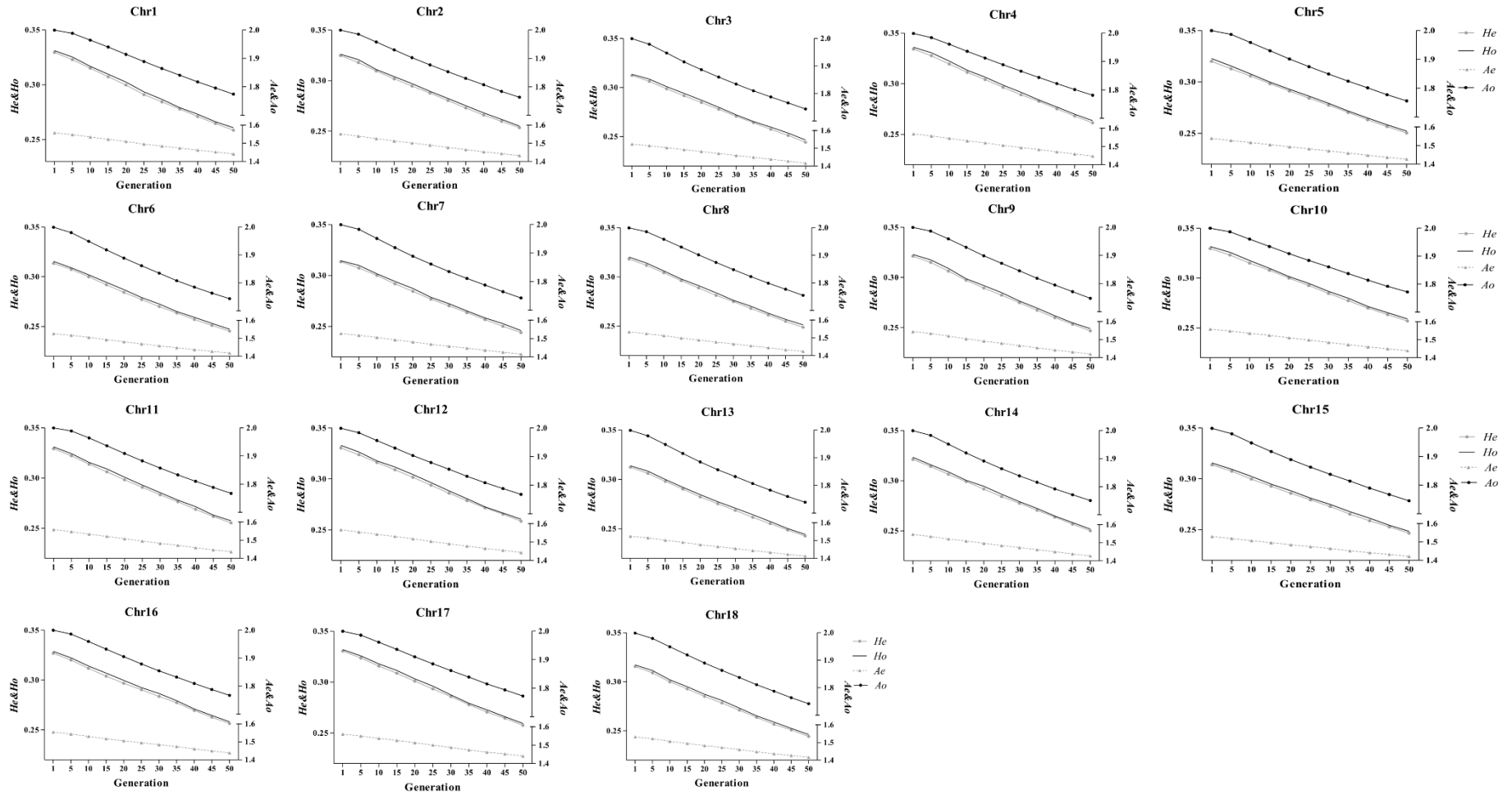

Supplement: Supplementary file 1 [file ajas-19-0035-suppl1.pdf]

Figure S2

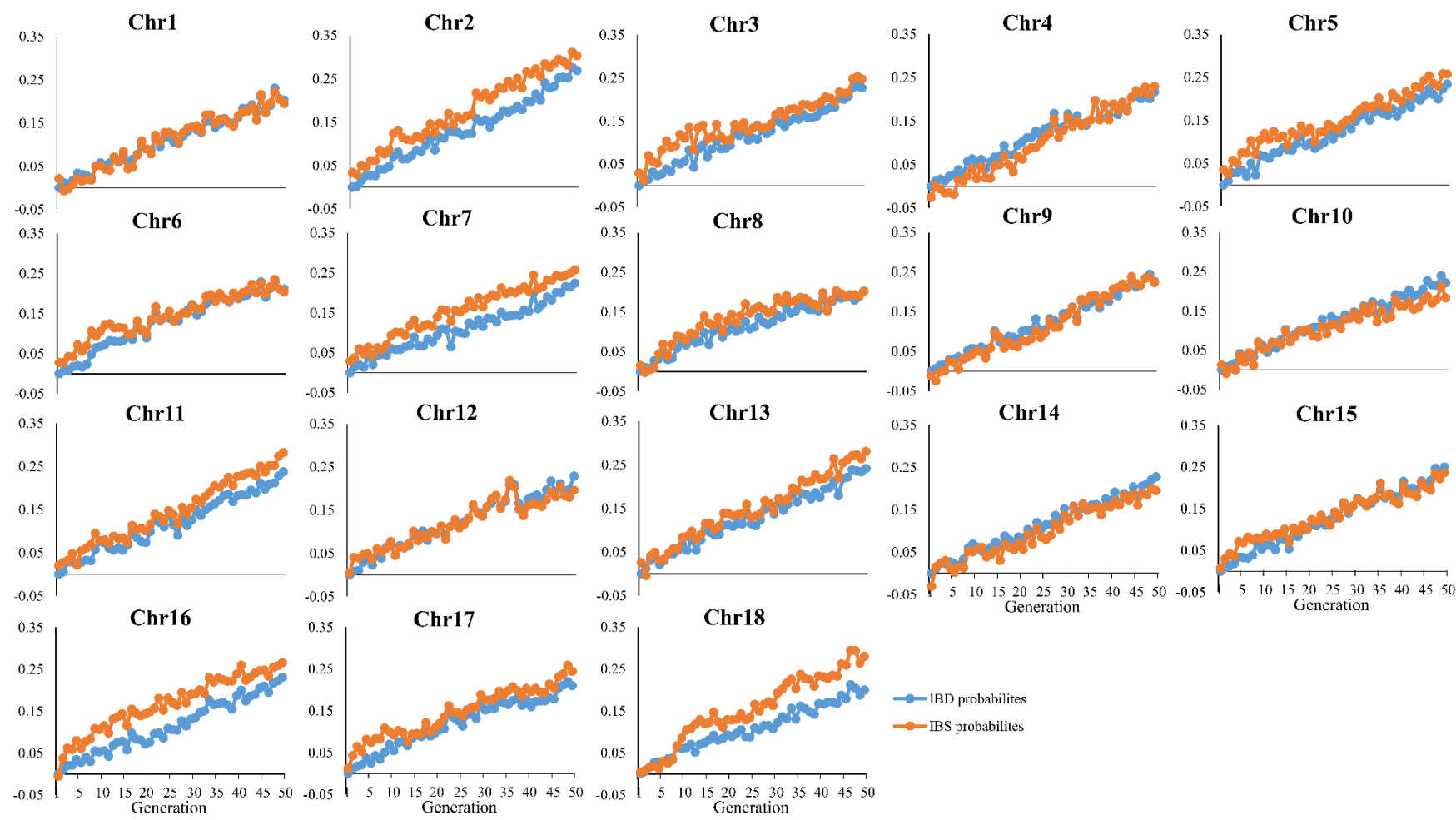

Supplement: Supplementary file 2 [file ajas-19-0035-suppl2.pdf]

Figure S3

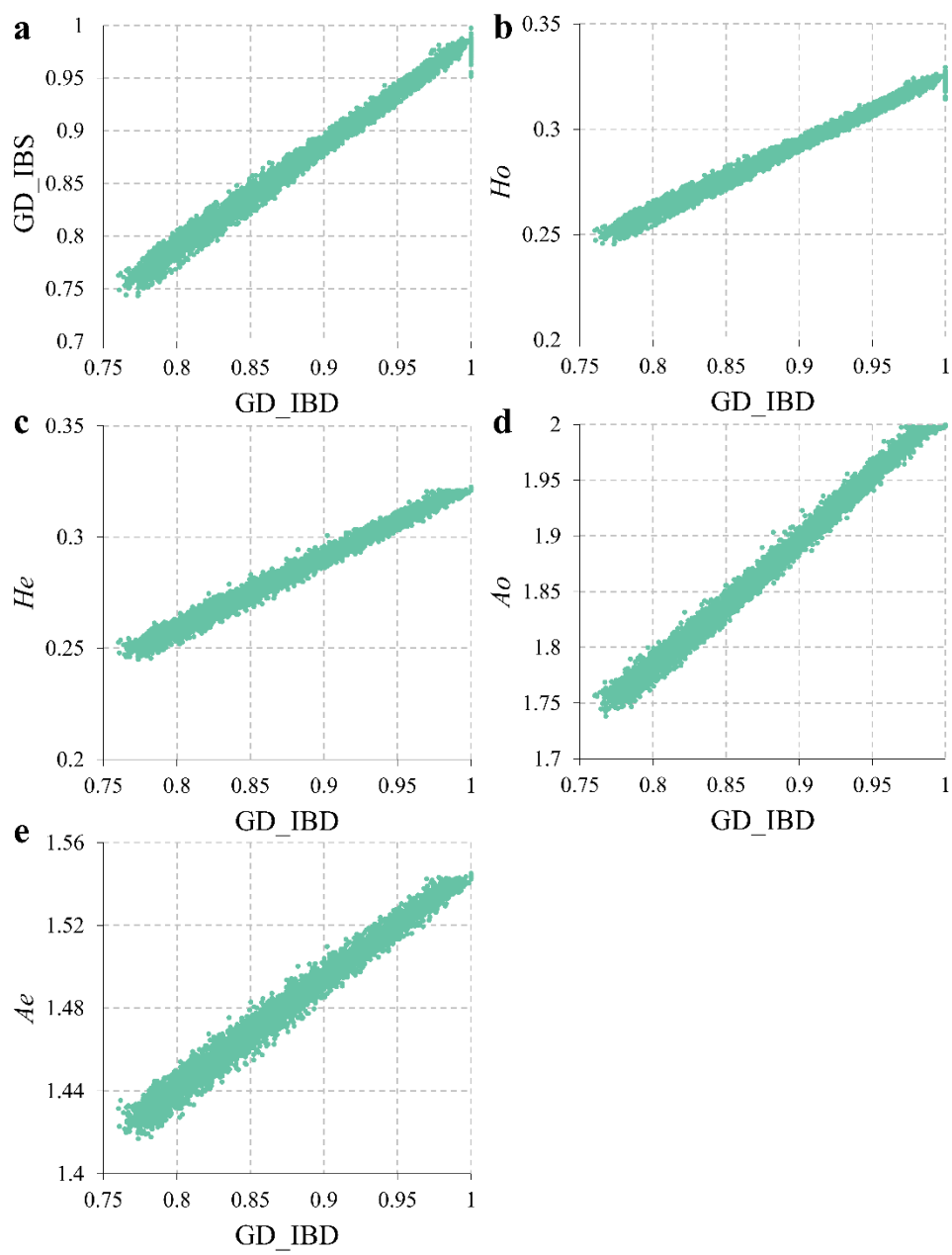

Supplement: Supplementary file 3 [file ajas-19-0035-suppl3.pdf]
