# Supplementary material for: A double-labeling marker-based method for estimating inbreeding and parental genomic components in a population under conservation
Source: Asian-Australas J Anim Sci. 2019 Jul 1;33(1):12–23. doi: 10.5713/ajas.19.0035 (PMC6946976; doi:10.5713/ajas.19.0035)
Supplement: Supplementary file 4 [file ajas-19-0035-suppl4.pdf]

Table S1 The temporal changes of genomic contents of each male family

ID / *t*	M1	M2	M3	M4	M5	M6	M7	M8	M9	M10	M11	M12	M13	M14	M15	M16	M17	M18	M19	M20
1	0	0	0	0	0	0	0	0	0	0	0	0	0	0	0	0	0	0	0	0
2	0.0146	-0.0636	-0.0128	0.1764	-0.0992	0.1459	-0.1526	-0.137	-0.0339	-0.0811	0.0788	-0.1309	0.0355	0.071	-0.0165	0.144	-0.0212	0.0049	-0.0828	0.0189
3	0.091	-0.1996	-0.1769	0.2619	-0.1984	0.2854	-0.3686	-0.2993	-0.4025	-0.1864	0.2878	-0.4075	0.2258	0.361	0.2434	0.6443	0.1604	0.0614	-0.3292	0.2261
4	-0.0526	-0.1779	-0.2644	0.4372	-0.0648	0.1809	-0.5272	-0.0675	-0.3991	-0.1503	0.1471	-0.5544	0.2423	0.2769	0.187	0.7258	0.523	0.1604	-0.4388	0.1218
5	-0.1701	-0.1657	-0.313	0.7388	-0.236	0.1736	-0.5228	0.0442	-0.3432	-0.1791	0.1073	-0.5211	0.5373	0.1717	0.1436	0.749	0.5109	0.0607	-0.547	0.1482
6	-0.1599	-0.1533	-0.1239	0.8765	-0.3216	0.261	-0.5544	0.1254	-0.2733	-0.1093	0.0226	-0.5812	0.5145	0.206	0.0786	0.6679	0.558	-0.0198	-0.5295	0.2093
7	-0.0723	-0.085	-0.1057	0.7712	-0.281	0.2457	-0.555	0.0579	-0.3069	-0.1104	0.0558	-0.592	0.562	0.2024	0.1039	0.567	0.5401	0.0134	-0.4892	0.1995
8	-0.1187	-0.1194	-0.1185	0.8536	-0.3552	0.352	-0.5274	0.0599	-0.2991	-0.1366	0.0134	-0.5771	0.5196	0.2888	0.1502	0.5637	0.4582	-0.0034	-0.504	0.3022
9	-0.0779	-0.1942	-0.1675	0.8862	-0.3606	0.267	-0.5136	0.0488	-0.2612	-0.125	0.0017	-0.576	0.4571	0.3993	0.1103	0.5833	0.4233	-0.0045	-0.4442	0.3186
10	0.0048	-0.2709	-0.0833	0.9552	-0.3544	0.255	-0.5026	0.0668	-0.2507	-0.0931	-0.0279	-0.5961	0.4664	0.4549	0.1043	0.6128	0.4072	0.0006	-0.4456	0.3627
11	0.0053	-0.2368	-0.0685	1.0037	-0.3445	0.3515	-0.5961	0.0654	-0.231	-0.1199	-0.0388	-0.5884	0.4877	0.4664	0.1127	0.5522	0.4189	-0.0757	-0.3552	0.3709
12	0.0694	-0.2318	-0.1821	0.9734	-0.4215	0.3972	-0.6326	0.0616	-0.2383	-0.0418	-0.0125	-0.5588	0.466	0.4401	0.0243	0.5886	0.4037	-0.0563	-0.4035	0.3574
13	0.0502	-0.1925	-0.2049	0.9714	-0.4304	0.4756	-0.6383	0.0678	-0.213	-0.0442	-0.042	-0.5597	0.4091	0.4491	0.0173	0.5645	0.3707	-0.05	-0.4173	0.3016
14	0.1018	-0.2223	-0.2147	1.0029	-0.4181	0.5229	-0.6127	0.0247	-0.206	0.0103	-0.1136	-0.5436	0.3616	0.4336	0.0209	0.4851	0.397	-0.06	-0.4021	0.297
15	0.1113	-0.2204	-0.2203	1.0391	-0.4232	0.4757	-0.5947	0.0044	-0.217	0.038	-0.1405	-0.5521	0.3577	0.4455	0.1045	0.4746	0.4296	-0.0437	-0.4517	0.2264
16	0.05	-0.1928	-0.1937	1.0583	-0.3885	0.4656	-0.6128	0.0023	-0.2178	0.1276	-0.1084	-0.5669	0.3517	0.3886	0.1625	0.536	0.4536	-0.0639	-0.4724	0.2846
17	0.1114	-0.2257	-0.1949	1.1038	-0.3338	0.4516	-0.5883	0.005	-0.2619	0.1214	-0.1116	-0.5653	0.3503	0.4185	0.1648	0.4952	0.4218	-0.022	-0.5241	0.2913
18	0.028	-0.1976	-0.1947	1.0223	-0.3301	0.4682	-0.5693	0.087	-0.2652	0.1369	-0.0702	-0.588	0.3716	0.4465	0.1784	0.4702	0.4565	-0.0475	-0.5555	0.3367
19	-0.0012	-0.1919	-0.2006	0.9911	-0.3315	0.4292	-0.5532	0.0715	-0.238	0.1956	-0.0777	-0.5537	0.4269	0.3941	0.1606	0.4983	0.4732	-0.034	-0.5729	0.2983
20	0.0129	-0.181	-0.1988	0.9665	-0.317	0.4709	-0.5419	0.0868	-0.2117	0.2583	-0.0923	-0.5564	0.4284	0.3543	0.1024	0.4724	0.4864	-0.0377	-0.5719	0.2799
21	-0.0127	-0.2113	-0.1793	1.0338	-0.3543	0.4406	-0.542	0.1139	-0.2588	0.2472	-0.0879	-0.5866	0.3928	0.423	0.1044	0.4482	0.5696	-0.037	-0.5698	0.2994
22	-0.04	-0.2095	-0.1675	1.1104	-0.3653	0.5109	-0.5716	0.1264	-0.2754	0.1733	-0.089	-0.5788	0.4107	0.398	0.0573	0.4605	0.5676	-0.0639	-0.5532	0.2965
23	-0.0397	-0.245	-0.1685	1.1933	-0.3964	0.5011	-0.5949	0.1421	-0.2554	0.1649	-0.0691	-0.5717	0.4427	0.4381	0.0465	0.4453	0.6328	-0.069	-0.5787	0.2917
24	-0.0534	-0.2374	-0.1535	1.1717	-0.3884	0.4689	-0.6202	0.1325	-0.2621	0.152	-0.0638	-0.581	0.4364	0.4749	0.0596	0.4211	0.6075	-0.0788	-0.5849	0.2923
25	-0.1146	-0.2185	-0.193	1.2081	-0.3486	0.4461	-0.6521	0.1754	-0.2483	0.1195	-0.1093	-0.5655	0.4142	0.4898	0.0312	0.4433	0.6614	-0.1064	-0.5565	0.3197
26	-0.1226	-0.2414	-0.1857	1.2118	-0.3284	0.4544	-0.6504	0.1211	-0.2575	0.148	-0.1278	-0.5845	0.4134	0.4359	0.0433	0.3972	0.647	-0.0567	-0.5484	0.343
27	-0.0987	-0.2419	-0.206	1.1804	-0.3371	0.3992	-0.6672	0.1199	-0.2837	0.127	-0.1009	-0.6028	0.4031	0.4725	0.0398	0.4243	0.6025	-0.0204	-0.5166	0.3637
28	-0.1081	-0.246	-0.2492	1.2054	-0.315	0.3704	-0.6708	0.1007	-0.3027	0.0981	-0.0926	-0.5761	0.4116	0.4751	0.0446	0.4345	0.5289	-0.024	-0.5115	0.3889
29	-0.12	-0.2298	-0.252	1.1534	-0.2998	0.3366	-0.6759	0.1198	-0.3133	0.0537	-0.1018	-0.5989	0.4194	0.4792	0.0804	0.4641	0.5187	-0.0275	-0.5169	0.3981
30	-0.1123	-0.2853	-0.2772	1.1589	-0.2765	0.353	-0.6939	0.1296	-0.3208	0.062	-0.1088	-0.5987	0.4408	0.4787	0.0999	0.4538	0.4883	-0.0111	-0.4993	0.431
31	-0.0952	-0.2876	-0.2884	1.1898	-0.3074	0.3413	-0.6862	0.0601	-0.31	0.0576	-0.1179	-0.6021	0.4578	0.4905	0.1476	0.4141	0.4772	0.0242	-0.4918	0.4237

32	-0.112	-0.3018	-0.3315	1.1589	-0.2848	0.3551	-0.6767	0.0788	-0.3286	0.0944	-0.1151	-0.6098	0.4889	0.4675	0.1328	0.3887	0.476	0.0203	-0.4809	0.4162
33	-0.1295	-0.2929	-0.3506	1.1552	-0.2603	0.3839	-0.6679	0.0883	-0.3534	0.1235	-0.1145	-0.6349	0.471	0.4432	0.0874	0.3299	0.4943	0.0123	-0.4617	0.4382
34	-0.1154	-0.3116	-0.3187	1.1841	-0.286	0.3634	-0.6579	0.0927	-0.3776	0.155	-0.0897	-0.6195	0.4271	0.4237	0.101	0.2694	0.5194	-0.0243	-0.4648	0.4448
35	-0.1007	-0.3078	-0.3574	1.238	-0.313	0.3398	-0.6585	0.0813	-0.3903	0.1674	-0.1242	-0.6168	0.3924	0.4291	0.1244	0.2601	0.51	-0.019	-0.4709	0.4454
36	-0.1414	-0.2959	-0.3429	1.2305	-0.3325	0.3823	-0.649	0.039	-0.3707	0.1811	-0.1036	-0.6235	0.3605	0.4741	0.1561	0.2781	0.5317	-0.0215	-0.4676	0.4401
37	-0.1424	-0.3291	-0.3452	1.1944	-0.3164	0.3838	-0.6406	0.0939	-0.3729	0.1955	-0.1264	-0.617	0.3286	0.4299	0.1686	0.3314	0.5526	-0.0404	-0.4717	0.445
38	-0.1143	-0.328	-0.3417	1.2307	-0.3069	0.4157	-0.6374	0.0594	-0.3392	0.2179	-0.1321	-0.5957	0.3583	0.4501	0.1943	0.2561	0.5561	-0.0674	-0.4741	0.4743
39	-0.0916	-0.3229	-0.3492	1.1667	-0.3118	0.4291	-0.6503	0.0527	-0.3387	0.2599	-0.1526	-0.6006	0.3501	0.4485	0.1813	0.2495	0.5887	-0.0561	-0.4611	0.4844
40	-0.1006	-0.3218	-0.3464	1.1726	-0.3492	0.4343	-0.6623	0.0489	-0.3627	0.2837	-0.1641	-0.5657	0.3526	0.4621	0.1641	0.2476	0.6066	-0.0322	-0.4571	0.457
41	-0.0973	-0.3002	-0.3414	1.1835	-0.3781	0.4278	-0.6896	0.0637	-0.369	0.2928	-0.1752	-0.568	0.3717	0.4557	0.1794	0.2563	0.6065	-0.0174	-0.4346	0.4381
42	-0.0546	-0.3097	-0.3269	1.1993	-0.3615	0.4491	-0.7087	0.0665	-0.371	0.2964	-0.1654	-0.5791	0.3689	0.4448	0.1619	0.2742	0.6083	-0.0419	-0.4424	0.4184
43	-0.0935	-0.3173	-0.3358	1.2047	-0.3501	0.4713	-0.6996	0.0864	-0.364	0.2887	-0.1573	-0.5743	0.3446	0.47	0.1783	0.2968	0.5901	-0.0584	-0.454	0.4067
44	-0.0997	-0.3024	-0.3259	1.1839	-0.3553	0.4738	-0.7042	0.064	-0.3749	0.3346	-0.1481	-0.5924	0.3388	0.4568	0.1935	0.3052	0.5774	-0.054	-0.4285	0.3787
45	-0.1326	-0.2872	-0.3471	1.2019	-0.3405	0.4747	-0.6922	0.0353	-0.3402	0.3437	-0.1604	-0.5889	0.3225	0.458	0.1671	0.2954	0.5334	-0.0543	-0.4148	0.401
46	-0.1198	-0.2821	-0.3718	1.1901	-0.3483	0.4762	-0.6934	0.0044	-0.3504	0.373	-0.1515	-0.5879	0.3532	0.4535	0.1326	0.3201	0.5334	-0.0815	-0.4156	0.4508
47	-0.0828	-0.294	-0.3975	1.1922	-0.3591	0.4538	-0.6888	-0.0002	-0.353	0.3716	-0.1381	-0.6065	0.3258	0.4685	0.1034	0.318	0.5533	-0.0771	-0.4306	0.4848
48	-0.1095	-0.2885	-0.4212	1.2164	-0.3686	0.4398	-0.697	-0.002	-0.3659	0.408	-0.1356	-0.6056	0.2876	0.4468	0.0968	0.3137	0.5763	-0.0831	-0.4269	0.4933
49	-0.0989	-0.2875	-0.4527	1.2323	-0.3791	0.436	-0.7202	0.0016	-0.3673	0.4842	-0.1468	-0.6088	0.2915	0.4792	0.1	0.3237	0.5858	-0.0831	-0.4341	0.5214
50	-0.0844	-0.3016	-0.4546	1.2268	-0.4108	0.4234	-0.718	0.0003	-0.3673	0.49	-0.1782	-0.6058	0.2997	0.484	0.1206	0.2906	0.5674	-0.0699	-0.4488	0.5296
