# Supplementary material for: A double-labeling marker-based method for estimating inbreeding and parental genomic components in a population under conservation
Source: Asian-Australas J Anim Sci. 2019 Jul 1;33(1):12–23. doi: 10.5713/ajas.19.0035 (PMC6946976; doi:10.5713/ajas.19.0035)
Supplement: Supplementary file 5 [file ajas-19-0035-suppl5.pdf]

Fortran Source Code

##aa_ana.for

```
      subroutine aa_ana

        implicit none

          common/resultaa01/fmaa
          common/resultaa02/ffaa

          common /popmaa/snpmaa
          common /popfaa/snpfaa

      ! this block is connected with main and r02 block

        integer,parameter::nc=19        !number of chromosomes
        integer,parameter::nl=1000      !number of marker
        integer,parameter::nmale=20 !number of male
        integer,parameter::nfemale=100 !number of female

        integer,dimension(nmale,nc,2,nl)::snpmaa
        integer,dimension(nfemale,nc,2,nl)::snpfaa

        integer,dimension(nc,nl,2,nmale)::maanl
        integer,dimension(nc,nl,2,nfemale)::faanl

          integer,dimension(nl,2,nmale)::inmaa
          integer,dimension(nl,2,nfemale)::infaa

          integer,dimension(nmale)::maa      !aa porpotion
          integer,dimension(nfemale)::faa

          real,dimension(nmale)::fmaa      !aa porpotion
          real,dimension(nfemale)::ffaa


          integer code,code2,temp
          integer i,ii,iii,j

          real temp1

     !+++++transfer block +++++++++++++++++++++++++++++++

        do iii=1,nc
           do ii=1,nl
           do i=1,2
           do j=1,nmale
             code2=snpmaa(j,iii,i,ii)
             maanl(iii,ii,i,j)=code2
           end do
           end do
           end do
          do ii=1,nl
          do i=1,2
          do j=1,nfemale
             code2=snpfaa(j,iii,i,ii)
             faanl(iii,ii,i,j)=code2
           end do
           end do
           end do
        end do
```

```
!------------- the calculation block -----------

maa=0;faa=0

do iii=1,nc ! number of chromsome

   do ii=1,nl !number of SNP each chromsome
   do i=1,2
   do j=1,nmale
     inmaa(ii,i,j)=maanl(iii,ii,i,j)
   end do
   end do
   end do

   do ii=1,nl
   do i=1,2
   do j=1,nfemale
     infaa(ii,i,j)=faanl(iii,ii,i,j)
   end do
   end do
   end do !end of nl cycle

!++++++++++++++++++++++++++++++++++++++++++++++++++
!++++++++++++++++++++++++++++++++++++++++++++++++++

  do ii=1,nl
  do i=1,2

  do j= 1,nmale
     code=inmaa(ii,i,j)
     if(code<=nmale)then
        temp=maa(code)
        temp=temp+1
        maa(code)=temp
     else
        code2=code-100
        temp=faa(code2)
        temp=temp+1
        faa(code2)=temp
     end if
  end do ! end of male

  do j= 1,nfemale
     code=infaa(ii,i,j)
     if(code<=nmale)then
        temp=maa(code)
        temp=temp+1
        maa(code)=temp
     else
        code2=code-100
        temp=faa(code2)
        temp=temp+1
        faa(code2)=temp
     end if
   end do !end of female

   end do !end of i cycle
   end do !end of a given chromsome
end do !end of a set of chromsome

! -------------------------------------
  do i=1,nmale
     temp=maa(i)
```

```
            temp1=float(temp)/float(nc*nl*6)-1.0
            fmaa(i)=temp1
          end do
          do i=1,nfemale
            temp=faa(i)
            temp1=float(temp)/(float(nc*nl)+float(nc*nl)/5.0)-1.0
            ffaa(i)=temp1
          end do

         end subroutine aa_ana
```

##aa_mark.for

```
         subroutine aa_mark

          implicit none

           common /popmaa/snpmaa
           common /popfaa/snpfaa

          integer,parameter::nc=19         !number of chromosomes
          integer,parameter::nl=1000       !number of marker
          integer,parameter::nmale=20 !number of male
          integer,parameter::nfemale=100 !number of female

          integer,dimension(nmale,nc,2,nl)::snpmaa
          integer,dimension(nfemale,nc,2,nl)::snpfaa

           integer i,ii,iii,j

        !--------ancesotr code for base poulation -----------------------
           snpmaa=0
           snpfaa=0

               do j=1,nmale
                 do  iii=1,nc
                 do  ii=1,2
                 do  i=1,nl
                   snpmaa(j,iii,ii,i)=j
                 end do
                 end do
                 end do
               end do

               do j=1,nfemale
                 do  iii=1,nc
                 do  ii=1,2
                 do  i=1,nl
                   snpfaa(j,iii,ii,i)=j+100
                 end do
                 end do
                 end do
               end do

         end subroutine aa_mark
```

##aa_num.for

```
          subroutine aa_num(code)

             implicit none

             integer code
```

```
        select case(code)
              case(1)
                k1=k1+1
              case(2)
                k2=k2+1
                case(3)
                k3=k3+1
              case(4)
                k4=k4+1
              case(5)
                k5=k5+1
              case(6)
                k6=k6+1
                case(7)
                k7=k7+1
              case(8)
                k8=k8+1
                case(9)
                k9=k9+1
              case(10)
                k10=k10+1
                case(11)
                k11=k11+1
              case(12)
                k12=k12+1



          end select

##ancestor.for
      subroutine ancestor

            implicit none

          common /codmale01/codmchange
          common /codpfemale01/codfchange

              common/an01/comam
          common/an02/comaf    ! form main block


        integer,parameter::nc=19        !number of chromosomes
        integer,parameter::nl=1000      !number of marker
        integer,parameter::nmale=10 !number of male
        integer,parameter::nfemale=50 !number of female
        integer,dimension(nmale,nc,2,nl)::codmchange
        integer,dimension(nfemale,nc,2,nl)::codfchange

        ! this is for calculation based on SNP loci

        integer,dimension(nmale)::comam
        integer,dimension(nfemale)::comaf
        integer,dimension(nl)::cham      !common ancestor for given chromsome
        integer,dimension(nl)::chaf

        integer,dimension(nc,2,nl)::inam !common ancestor for given individual
        integer,dimension(nc,2,nl)::inaf
        integer,dimension(nmale,nc,nmale)::popam !common a for pop
        integer,dimension(nfemale,nc,nfemale)::popaf
```

```
integer temp, code ! inter-variable
   integer i,ii,iii,j
   integer i1,icom

!------------ the calculation block for ancestor comparpent -----------

   popam=0; popaf=0

   do j=1,nmale !
      inam=0;inaf=0
    do iii=1,nc ! number of chromsome
      cham=0; chaf=0
   do ii=1,2
   do i=1,nl

      temp=codmchange(j,iii,ii,i)

        if(temp<100) then
        do i1=1,nmale
          code=comam(i1)
          icom=i1
          if(temp==code) exit
            end do
        cham(icom)=cham(icom)+1
      else
        do i1=1,nfemale
            code=comaf(i1)
            icom=i1
          if(temp==code) exit
            end do
        chaf(icom)=chaf(icom)+1
    end if
   end do

   end do
      inam(iii,1:nmale)=cham
      inaf(iii,1:nfemale)=chaf
   end do
      popam(j,1:nc,1:nmale)=inam
      popaf(j,1:nc,1:nfemale)=inaf
   end do

! the block of calculation the ancestor frequency

  end subroutine ancestor
```

##normal_distribution02.for

```
   subroutine normal_distribution02

   implicit none

    common /comseed/seed
    common /mutation02/number_mutation !connected with mutation block

    real,parameter::pi=3.14
    real,parameter::u=2.5e-6      ! mutation rate
    integer,parameter::nc=19      !number of chromosomes
    integer,parameter::nl=1000    !number of marker
    integer,parameter::nmale=20        !number of male
    integer,parameter::nfemale=100 !number of male
    integer number_mutation

    real r,r1,r2,y1,y2
```

```
      real a,c,lambda

      integer,dimension(1):: seed
      integer i

       !number of mutation per chromsome per generation

     call random_seed(put=seed)

        i=-1;a=3
        lambda=2*(nmale+nfemale)*nc*u*nl

        do while(i<0)
          call random_number(r)
            r1=r
          call random_number(r)
            r2=r
          call random_number(r)
            y1=2*a*r1-a
          call random_number(r)
            y2=(1/sqrt(pi))*r2
            c=(1/sqrt(2*pi))*exp(-y1**2/2)
          if(y2<=c) i=1
        end do

        y2=lambda+sqrt(lambda)*y2
        number_mutation=int(y2)

     call random_seed(get=seed)

    end subroutine normal_distribution02

##poisson_process.for
     subroutine poisson_process

     implicit none

      common /comseed/seed
      common/cross/crossover !connected with reproduction block

      real,parameter::lambda=1.0
      integer,parameter::nc=19

      integer,dimension(1):: seed
      integer,dimension(nc)::crossover

      integer ii,i,x
      real m,r

      call random_seed(put=seed)

      do ii=1,nc

        i=-1; x=-1
        m=exp(lambda)

        do while(i<0)

          call random_number(r)
          m=m*r
          x=x+1
          if(m<1.0) i=1
        end do
```

```
                crossover(ii)=x
            end do

            call random_seed(get=seed)

          end subroutine poisson_process

##random_mating2011.for
            subroutine random_mating2011

           implicit none

            common /commonseed/seed
            common /femalerandom/female_num
            common /malerandom/male_num

            integer,parameter:: malepop=20,femalepop=100

            integer,dimension(femalepop)::female_num
            integer,dimension(malepop)::male_num

            integer i
            integer k,num
             real r


            integer,dimension(1):: seed

              call random_seed(put=seed)


             female_num=0;male_num=0

               k=0
             do while(k<=femalepop)

              call random_number(r)
              num=int(r*femalepop+1)
             do i=1,femalepop
              if(num==female_num(i)) exit
             end do

             if(i>=femalepop)   then
               k=k+1
               female_num(k)=num
             end if
            end do

            k=0
           do while(k<=malepop)
             call random_number(r)
              num=int(r*malepop+1)
             do i=1,malepop
              if(num==male_num(i)) exit
             end do

             if(i>=malepop)      then
               k=k+1
               male_num(k)=num
             end if
           end do

           call random_seed(get=seed)
```

```
      end    subroutine random_mating2011
```

##snp_ana.for

```
     subroutine snp_ana


      implicit none

      common /resultaa/aa
      common/rsnp_ana01/k1,k2,k3,k4,k5,k6,k7,k8,k9,k10,ko,kf

      integer,parameter::nc=19        !number of chromosomes
      integer,parameter::nl=1000    !number of marker

      real,dimension(nc,nl,2)::aa

      integer k1,k2,k3,k4,k5
      integer k6,k7,k8,k9,k10
      integer ko,kf

      real temp
      integer i,ii

    !++++++++snp frequency distribution    ++++++++++++++


       k1=0; k2=0; k3=0; k4=0; k5=0
       k6=0; k7=0; k8=0; k9=0; k10=0
       ko=0; kf=0

       do ii=1,nc
       do i=1,nl

          temp=aa(ii,i,1)

         if(0<=temp .and. temp <0.1) then
            k1=k1+1
         else if(0.1<=temp .and. temp <0.2) then
            k2=k2+1
         else if(0.2<=temp .and. temp <0.3) then
            k3=k3+1
         else if(0.3<=temp .and. temp <0.4) then
              k4=k4+1
         else if(0.4<=temp .and. temp <0.5) then
            k5=k5+1
         else if(0.5<=temp .and. temp <0.6) then
              k6=k6+1
         else if(0.6<=temp .and. temp <0.7) then
            k7=k7+1
         else if(0.7<=temp .and. temp <0.8) then
              k8=k8+1
         else if(0.8<=temp .and. temp <0.9) then
            k9=k9+1
         else if(0.9<=temp .and. temp <=1.0) then
            k10=k10+1
         end if

         if(temp<=0.0001) then
            ko=ko+1
         else if(temp>=0.9999) then
            kf=kf+1
```

```
        end if

      end do
      end do

     end subroutine snp_ana
```

##snp_count.for

```
     subroutine snp_count

      implicit none


        common /resultaa/aa ! connected with calculation subroutine

        common/resultcount01/gho,ga_rich
        common/resultcount02/ncho
        common/resultcount03/nca_rich

        common /popmale/ snpmale
        common /popfemale/ snpfemale

      integer,parameter::nc=19        !number of chromosomes
      integer,parameter::nl=1000    !number of marker
      integer,parameter::nmale=20 !number of male
      integer,parameter::nfemale=100 !number of female
      integer,dimension(nmale,nc,2,nl)::snpmale
      integer,dimension(nfemale,nc,2,nl)::snpfemale
      integer,dimension(nc,nl,2,nmale)::snpmnl
      integer,dimension(nc,nl,2,nfemale)::snpfnl
      ! this is for calculation based on SNP loci

      real,dimension(nc,nl,2)::aa            ! genic frequency for gonomic snp loci
      real,dimension(2)::fre_allele        ! allelic frequency for a given loci

        integer,dimension(nl,2,nmale)::indim
        integer,dimension(nl,2,nfemale)::indif

        real,dimension(nc)::ncho,nca_rich
        real ga_rich          ! allelic number
        real gho                 ! gonomic ho

        integer kk1,kk2 ! heterozygosity or homozygosity

        integer nchomo,ncheter
        ! homozygasity and heterzygosity for a given chromsome
        integer ghomo,gheter ! gonomic homozygasity and heterzygosity


        real h

        integer code,gnum,ncnum ! inter-variable
        real        tempfre,tempa

        integer i,ii,iii,j

     !+++++transfer block +++++++++++++++++++++++++++++++

      do iii=1,nc

         do ii=1,nl
         do i=1,2
         do j=1,nmale
```

```
      code=snpmale(j,iii,i,ii)
      snpmnl(iii,ii,i,j)=code
    end do
    end do
    end do
  do ii=1,nl
  do i=1,2
  do j=1,nfemale
      code=snpfemale(j,iii,i,ii)
      snpfnl(iii,ii,i,j)=code
    end do
    end do
    end do
end do ! the end of transfer block

!------------ the calculation block -----------
!+++++++++++++++++++++++++++++++++++++++++++++++++

ghomo=0; gheter=0; gnum=0
!gnum is number of genomic snp

  do iii=1,nc ! number of chromsome
      nchomo=0;ncheter=0;ncnum=0

  do ii=1,nl
  do i=1,2
  do j=1,nmale
    indim(ii,i,j)=snpmnl(iii,ii,i,j)
  end do
  end do
  end do

  do ii=1,nl
  do i=1,2
  do j=1,nfemale
    indif(ii,i,j)=snpfnl(iii,ii,i,j)
  end do
  end do
  end do

! the block of calculate the allele frequence

do ii=1,nl ! cycle of nl

  do i=1,2
        tempfre=aa(iii,ii,i)
        fre_allele(i)=tempfre

        if(tempfre>0.001) then
            gnum=gnum+1
            ncnum=ncnum+1
        end if
    end do

!___count the homozygasity and heterozygasity ___________

  do j= 1,nmale
    kk1=indim(ii,1,j)
    kk2=indim(ii,2,j)

    if(kk1==kk2) then
        ghomo=ghomo+1
        nchomo=nchomo+1
```

```
            else
               gheter=gheter+1
               ncheter=ncheter+1
            end if
         end do
         !++++++++++++++++++++++++++++++++
        do j= 1,nfemale
            kk1=indif(ii,1,j)
            kk2=indif(ii,2,j)

            if(kk1==kk2) then
               ghomo=ghomo+1
               nchomo=nchomo+1
            else
               gheter=gheter+1
               ncheter=ncheter+1
            end if
         end do

        end do !end of nl cycle

           h=float(ncheter)/float(ncheter+nchomo)
           ncho(iii)=h
           tempa=float(ncnum)/float(nl)
           nca_rich(iii)=tempa

        end do !end of nc cyclue (chromsome)

           gho=float(gheter)/float(gheter+ghomo)
           ga_rich=float(gnum)/float(nl*nc)

        end subroutine snp_count
```

##snp_diversity.for

```
            subroutine snp_diversity


            implicit none

            common /resultaa/aa
            common/resultdiv2/mean_He,mean_allele,mean_ae,mean_p,num_p
            common/resultdiv21/num_rare1,num_rare2

            integer,parameter::nc=19        !number of chromosomes
            integer,parameter::nl=1000    !number of marker

            real,dimension(nc,nl,2)::aa
            real,dimension(2)::freq_allele
            integer,dimension(nc,nl)::num_allele !allelic number
            real ,dimension(nc,nl)::Ae,He,p         ! P is portion of polymorphic loci
            real mean_allele,mean_He,mean_ae,mean_p,num_p
            !num_p is snp locci

            integer ii,i,j

            integer numtemp,ni
            real temp,temp2
            real tempae,tempHe,tempp

            integer num_rare1,num_rare2
            integer temp_rare1,temp_rare2
```

```
!----------- the start of calculation    ----------------

       ni=0; tempHe=0; temp2=0; tempp=0
       num_allele=0;ae=0;he=0;p=0
       num_rare1=0;num_rare2=0

       do ii=1,nc
       do i=1,nl
          numtemp=0; tempae=0
          temp_rare1=0;temp_rare2=0

        do j=1,2
           freq_allele(j)=aa(ii,i,j)
           temp=freq_allele(j)

           tempae=tempae+temp*temp ! effective allelic rich

             if(temp>=0.001) then
                numtemp=numtemp+1              ! allelic number
             end if

           if(0.001<=temp.and.temp<0.02) then
                temp_rare1=temp_rare1+1 ! rare allele
             end if

           if(0.001<=temp.and.temp<0.05) then
                temp_rare2=temp_rare2+1
             end if
        end do ! end of j cycle

           num_allele(ii,i)=numtemp        ! allelic number of given loci
           Ae(ii,i)=1.0/tempae
           He(ii,i)=1-tempae
           ni=ni+numtemp                ! total allelic number of gonomics

           tempHe=tempHe+(1-tempae)
           temp2=temp2+1.0/tempae
           num_rare1=num_rare1+temp_rare1
           num_rare2=num_rare2+temp_rare2

              if (numtemp>1) then
                p(ii,i)=1
                tempp=tempp+1
              else
                 p(ii,i)=0
              end if

             end do ! end of cycle snp loci in a given chromsome

          end do

          mean_allele=float(ni)/float(nc*nl) !ni is gonomic allelic number
          mean_He=tempHe/float(nc*nl)               !tempHe is gonomic he
          mean_ae=temp2/float(nc*nl)              !temp2 is gonomic ae
          mean_p=tempp/float(nc*nl)                     !tempp is gonomic pp
          num_p=tempp                                    !num_p is gonomic np


!-------------------------------------------------

       end subroutine snp_diversity
```

##snp_ibd.for

```
 subroutine snp_ibd

   implicit none

     common/resultibd01/ngibd,ngibs
     common/resultibd02/ncibd
     common/resultibd03/ncibs

     common /popmale/snpmale
     common /popfemale/snpfemale
     common /popmaa/snpmaa
     common /popfaa/snpfaa

     ! this block is connected with main and r02 block

   integer,parameter::nc=19        !number of chromosomes
   integer,parameter::nl=1000      !number of marker
   integer,parameter::nmale=20 !number of male
   integer,parameter::nfemale=100 !number of female
   integer,dimension(nmale,nc,2,nl)::snpmale
   integer,dimension(nfemale,nc,2,nl)::snpfemale
   integer,dimension(nmale,nc,2,nl)::snpmaa
   integer,dimension(nfemale,nc,2,nl)::snpfaa

   integer,dimension(nc,nl,2,nmale)::snpmnl
   integer,dimension(nc,nl,2,nfemale)::snpfnl
   integer,dimension(nc,nl,2,nmale)::maanl
   integer,dimension(nc,nl,2,nfemale)::faanl
   ! this is for calculation based on SNP loci

     integer,dimension(nl,2,nmale)::indim,inmaa
     integer,dimension(nl,2,nfemale)::indif,infaa

     real,dimension(nc)::ncibd,ncibs
     real ngibd,ngibs

     integer kk1,kk2      ! heterozygosity or homozygosity
     integer kkc1,kkc2 ! common code

     integer ng_homo,ng_heter
     integer nc_homo,nc_heter
     integer ncnum_ibd,ngnum_ibd


     integer code,code2
     integer i,ii,iii,j
     real h1,h2

!+++++transfer block ++++++++++++++++++++++++++++++

   do iii=1,nc
      do ii=1,nl
      do i=1,2
      do j=1,nmale
        code=snpmale(j,iii,i,ii)
        snpmnl(iii,ii,i,j)=code

        code2=snpmaa(j,iii,i,ii)
        maanl(iii,ii,i,j)=code2
      end do
      end do
      end do
     do ii=1,nl
```

```
    do i=1,2
    do j=1,nfemale
       code=snpfemale(j,iii,i,ii)
       snpfnl(iii,ii,i,j)=code

       code2=snpfaa(j,iii,i,ii)
       faanl(iii,ii,i,j)=code2
     end do
     end do
     end do
  end do
!------------------------------------------------
!------------- the calculation block -----------
!------------------------------------------------
  ng_homo=0;ng_heter=0
  ngnum_ibd=0

 do iii=1,nc ! number of chromsome
    nc_homo=0;nc_heter=0
    ncnum_ibd=0

    do ii=1,nl !number of SNP each chromsome
    do i=1,2
    do j=1,nmale
      indim(ii,i,j)=snpmnl(iii,ii,i,j)
      inmaa(ii,i,j)=maanl(iii,ii,i,j)
    end do
    end do

    do i=1,2
    do j=1,nfemale
      indif(ii,i,j)=snpfnl(iii,ii,i,j)
      infaa(ii,i,j)=faanl(iii,ii,i,j)
    end do
    end do
    end do
 ! the above block is in individual unit for SNP
 !++++++++++++++++++++++++++++++++++++++++++++++++++++
 ! the follow block is count for ibd

  do ii=1,nl
  do i=1,2

  do j= 1,nmale
     kk1=indim(ii,1,j)
     kk2=indim(ii,2,j)
     kkc1=inmaa(ii,1,j)
     kkc2=inmaa(ii,2,j)

     if(kk1==kk2) then
        nc_homo=nc_homo+1
        ng_homo=ng_homo+1
     else
        nc_heter=nc_heter+1
        ng_heter=ng_heter+1
     end if

     if(kk1==kk2.and.kkc1==kkc2) then
          ncnum_ibd=ncnum_ibd+1
          ngnum_ibd=ngnum_ibd+1
     end if
  end do
  !++++++++++++++++++++++++++++++++++++
```

```
        do j= 1,nfemale
            kk1=indif(ii,1,j)
            kk2=indif(ii,2,j)
            kkc1=infaa(ii,1,j)
            kkc2=infaa(ii,2,j)

            if(kk1==kk2) then
              nc_homo=nc_homo+1
              ng_homo=ng_homo+1
            else
              nc_heter=nc_heter+1
              ng_heter=ng_heter+1
            end if

            if(kk1==kk2.and.kkc1==kkc2) then
                ncnum_ibd=ncnum_ibd+1
                ngnum_ibd=ngnum_ibd+1
            end if

          end do ! end of female

          end do ! end of a snp locus(i cycle)
          end do !end of a chromsome

            h1=float(nc_homo)/float(nc_heter+nc_homo)
              ncibs(iii)=h1
            h2=float(ncnum_ibd)/float(nc_heter+nc_homo)
              ncibd(iii)=h2
        end do !end of cycle chromsome

          h1=float(ng_heter)/float(ng_heter+ng_homo)
            ngibs=h1
          h2=float(ngnum_ibd)/float(ng_heter+ng_homo)
            ngibd=h2

        end subroutine snp_ibd
```

##snp_input.for

```
        subroutine snp_input

            implicit none

          common /popmale/snpmale
          common /popfemale/snpfemale

          integer,parameter::nc=19        !number of chromosomes
          integer,parameter::nl=1000      !number of marker
          integer,parameter::nmale=20 !number of male
          integer,parameter::nfemale=100 !number of female
          integer,parameter::nloci=2*nc*nl

          integer,dimension(nmale,nc,2,nl)::snpmale
          integer,dimension(nfemale,nc,2,nl)::snpfemale

          integer,dimension(nc,nl,2,nmale)::snpmnl
          integer,dimension(nc,nl,2,nfemale)::snpfnl

          ! this is for calculation based on SNP loci

            integer,dimension(nloci,nmale)::locim
            integer,dimension(nloci,nfemale)::locif

            integer code,itemp
```

```
      integer i,ii,iii
      integer j,jj

    open(1,file='f:\database\mnlsnp.txt')
    open(2,file='f:\database\fnlsnp.txt')

    read(1,*) ((locim(j,jj),jj=1,nmale),j=1,nloci)
    read(2,*) ((locif(j,jj),jj=1,nfemale),j=1,nloci)
    close(1);close(2)
    ! the block is read snp_data ++++++++++++++++++++++
      itemp=1
        do iii=1,nc
        do ii=1,nl
        do i=1,2
        do j=1,nmale
          code=locim(itemp,j)
          snpmnl(iii,ii,i,j)=code
        end do
          itemp=itemp+1
        end do
        end do
        end do

        itemp=1
      do iii=1,nc
      do ii=1,nl
      do i=1,2
      do j=1,nfemale
          code=locim(itemp,j)
          snpfnl(iii,ii,i,j)=code
      end do
          itemp=itemp+1
      end do
      end do
      end do
! the snp_data transfer to snpmnl and snpnfnl
    !+++++++++++++++++++++++++++++++++++++++++++++++++++++++++++
    do iii=1,nc
        do ii=1,nl
        do i=1,2
        do j=1,nmale
          code= snpmnl(iii,ii,i,j)
          snpmale(j,iii,i,ii)=code
        end do
        end do
        end do
        end do

        do iii=1,nc
        do ii=1,nl
        do i=1,2
        do j=1,nfemale
          code= snpfnl(iii,ii,i,j)
          snpfemale(j,iii,i,ii)=code
        end do
        end do
        end do
        end do

    !#####################################################

    end subroutine snp_input
```

snp_m02.for

```
      subroutine snp_m02
    ! mutation subroutine

       implicit none

     common /comseed/seed

     common /popmale/snpmale
     common /popfemale/snpfemale

     common /mutation02/number_mutation !connected with mutation block

     integer,parameter::nc=19        !number of chromosomes
     integer,parameter::nl=1000      !number of marker
     integer,parameter::nmale=20 !number of male
     integer,parameter::nfemale=100 !number of male
     integer,dimension(nmale,nc,2,nl)::snpmale
     integer,dimension(nfemale,nc,2,nl)::snpfemale
     ! genetic structure of male and female population

     integer number_mutation,male_mutation,female_mutation
     integer imale,inc,inl,ia ! counters
     integer jj                   ! counters

     integer,dimension(1):: seed
     real r,temp
     integer code1

      external normal_distribution02

      call random_seed(put=seed)
      call normal_distribution02

        temp=float(nmale)/(float(nmale)+float(nfemale))
        temp=float(number_mutation)*temp
        male_mutation=int(temp)
        female_mutation=number_mutation-male_mutation


       do jj=1,male_mutation
         call random_number(r)
          imale=int(r*nmale+1)
         call random_number(r)
          inc=int(r*nc+1)
         call random_number(r)
          inl=int(r*nl+1)
         call random_number(r)
          ia=int(r*2+1)
         code1=snpmale(imale,inc,ia,inl)
         if(code1==1) then
          snpmale(imale,inc,ia,inl)=2
         else
          snpmale(imale,inc,ia,inl)=1
         end if

      end do

   !===mutation for female population==============================

       do jj=1,female_mutation
         call random_number(r)
```

```
              imale=int(r*nfemale+1)
            call random_number(r)
              inc=int(r*nc+1)
            call random_number(r)
              inl=int(r*nl+1)
            call random_number(r)
              ia=int(r*2+1)
            code1=snpfemale(imale,inc,ia,inl)
            if(code1==1) then
              snpfemale(imale,inc,ia,inl)=2
            else
              snpfemale(imale,inc,ia,inl)=1
            end if
!
          end do

        call random_seed(get=seed)


      end subroutine snp_m02
```

##snp_main.for

```
      program snp_main


      implicit none

      common /comseed/seed

      common /popmale/snpmale
      common /popfemale/snpfemale
      common /popmaa/snpmaa
      common /popfaa/snpfaa
     !this is for experiment under conservation
     !main block connected with calculation subroutine

      common/result1/ ngho,ngao1
      common/resultdiv2/nghe,ngao2,ngae,ngpp,ngnp
      common/resultdiv21/ngrare1,ngrare2
      !connected with snp_diversity subroutine

        common /rncdiver01/ncae    !ncdiver block
        common/rncdiver02/ncao
        common/rncdiver03/nche
        common/rncdiver04/ncnp
        common/rncdiver05/ncpp
        common/rncdiver06/ncrare1
        common/rncdiver07/ncrare2 !ncdiver block

        common/rsnp_ana01/k1,k2,k3,k4,k5,k6,k7,k8,k9,k10,ko,kf
        !connected with snp_ana subroutine

        common/resultcount01/gho01,gao01 !snp_count block
        common/resultcount02/ncho01
        common/resultcount03/ncao01

        common/resultibd01/ngibd,ngibs !snp_ibd block
        common/resultibd02/ncibd
        common/resultibd03/ncibs             !snp_ibd block

        common/resultaa01/fmaa    ! aa_ana block
        common/resultaa02/ffaa    ! aa_ana block
```

```
    integer,parameter::gi=50    ! number of mgeneration
    integer,parameter::rep=1             ! nember of replication

    integer,parameter::nc=19          !number of chromosomes
    integer,parameter::nl=1000      !number of marker
    integer,parameter::nmale=20    !number of male
    integer,parameter::nfemale=100 !number of female

    integer,dimension(nmale,nc,2,nl)::snpmale
    integer,dimension(nfemale,nc,2,nl)::snpfemale
    ! genetic snp-code of male and female population

    integer,dimension(nmale,nc,2,nl)::snpmaa
    integer,dimension(nfemale,nc,2,nl)::snpfaa
    ! ancestor code for base population

    real ngho,nghe,ngao1,ngao2,ngae,ngpp,ngnp
    integer ngrare1,ngrare2

    real,dimension(nc)::ncae,ncao,nche,ncnp,ncpp
    real,dimension(nc)::ncrare1,ncrare2
    integer k1,k2,k3,k4,k5
    integer k6,k7,k8,k9,k10
    integer ko,kf

    real gho01,gao01
    real,dimension(nc)::ncho01,ncao01

    real,dimension(nmale)::fmaa      ! aa_ana block
    real,dimension(nfemale)::ffaa

    real ngibd,ngibs,ibsbase                              !ibd block
    real,dimension(nc):: ncibd,ncibs,ncibsbase

    integer,dimension(1):: seed
    integer count

    integer t ! t=counter of generation
    integer i,ii,iii
    integer jj,j1,j2,code

    integer,dimension(nc,2,nl)::inmaa
    integer,dimension(nl,2)::ncmaa
    real temp,temp1,temp2


      external snp_input
      external snp_marker
      external snp_m02
      external snp_r02
      external snp_ibd
      external aa_ana

      external snp_diversity
      external snp_calculaion
      external snp_ana
      external snp_count
      external snp_ncdive

!++++++++++++++++++++++++++++++++++++++++++++++
      call system_clock(count)
          seed=count
```

```
      call snp_input
!+++++++++++++++++++++++++++++++++++++++++++ +++++++++
         call snp_calculation
            call snp_diversity
         call snp_ana
         call snp_ncdiv
         call snp_count

    open(11,file='c:\dataresult\hobase01.txt')
     write(11,101) ngho,ngao1
     ibsbase=ngho
    open(12,file='c:\dataresult\diverbase02.txt')
     write(12,102) nghe,ngao2,ngae,ngpp,ngnp
     write(12,103) ngrare1,ngrare2

    open(14,file='c:\dataresult\diverbase03.txt')
         write(14,104) k1,k2,k3,k4,k5,k6,k7,k8,k9,k10,ko,kf
    open(15,file='c:\dataresult\diverbase05.txt')
     write(15,105) (ncae(ii),ii=1,nc)
     write(15,*)
     write(15,105) (ncao(ii),ii=1,nc)
     write(15,*)
     write(15,105) (nche(ii),ii=1,nc)
     write(15,*)
     write(15,105) (ncnp(ii),ii=1,nc)
     write(15,*)
     write(15,105) (ncpp(ii),ii=1,nc)
     write(15,*)
     write(15,105) (ncrare1(ii),ii=1,nc)
     write(15,*)
     write(15,105) (ncrare2(ii),ii=1,nc)
     write(15,*)
     write(15,105) (ncho01(ii),ii=1,nc)
     write(15,*)
     write(15,105) (ncao01(ii),ii=1,nc)

    close(11);close(12);close(13);close(14);close(15)

    open(16,file='c:\dataresult\maa001.txt')
    open(17,file='c:\dataresult\faa001.txt')
    open(51,file='c:\dataresult\51.txt')
    open(52,file='c:\dataresult\52.txt')
    open(53,file='c:\dataresult\53.txt')

    call aa_mark
    call aa_ana
     write (16,106) (fmaa(ii),ii=1,nmale)
     write (17,107) (ffaa(ii),ii=1,nfemale)
    close(16);close(17)
    call      snp_ibd
              ngibs=1-(ngibs/ibsbase)
         write (51,101) ngibd,ngibs
         write (52,105) (ncibd(ii),ii=1,nc)
         write (53,105) (ncibs(ii),ii=1,nc)
         ncibsbase=ncibs
    close(51);close(52);close(53)


! ====== the block above is basepopulation =====================
  open(21,file='c:\dataresult\01.txt')
  open(22,file='c:\dataresult\02.txt')
  open(23,file='c:\dataresult\03.txt')
  open(24,file='c:\dataresult\04.txt')
```

```
open(25,file='c:\dataresult\05.txt')
open(26,file='c:\dataresult\06.txt')
open(27,file='c:\dataresult\07.txt')
open(28,file='c:\dataresult\08.txt')
open(29,file='c:\dataresult\09.txt')

open(30,file='c:\dataresult\10.txt')
open(31,file='c:\dataresult\11.txt')
open(32,file='c:\dataresult\12.txt')
open(33,file='c:\dataresult\13.txt')
open(34,file='c:\dataresult\14.txt')
open(35,file='c:\dataresult\15.txt')
open(36,file='c:\dataresult\16.txt')

open(40,file='c:\dataresult\40.txt')
open(41,file='c:\dataresult\41.txt')
open(42,file='c:\dataresult\42.txt')

open(50,file='c:\dataresult\50.txt')


 do i=1,rep

    print*,i

    call snp_input
    call aa_mark

 do t=1,gi

      call snp_m02
      call snp_r02

      call snp_calculation
        write(21,101) ngho,ngao1

      call snp_diversity
        write(22,102) nghe,ngao2,ngae,ngpp,ngnp
        write(29,103) ngrare1,ngrare2
      call snp_ibd
              ngibs=1-(ngibs/ibsbase)
      write (23,101) ngibd,ngibs
              do ii=1,nc
                  temp1=ncibsbase(ii)
                  temp2=ncibs(ii)
                  temp=1-(temp2/temp1)
                  ncibs(ii)=temp
              end do
      write (24,105) (ncibd(ii),ii=1,nc)
      write (25,105) (ncibs(ii),ii=1,nc)

      call aa_ana

      write (26,106) (fmaa(ii),ii=1,nmale)
      write (27,107) (ffaa(ii),ii=1,nfemale)

          call snp_ana
      write(28,104) k1,k2,k3,k4,k5,k6,k7,k8,k9,k10,ko,kf

      call snp_ncdiv
        write(30,105) (ncae(ii),ii=1,nc)
        write(31,105) (ncao(ii),ii=1,nc)
        write(32,105) (nche(ii),ii=1,nc)
```

```
      write(33,105) (ncnp(ii),ii=1,nc)
      write(34,105) (ncpp(ii),ii=1,nc)
      write(35,105) (ncrare1(ii),ii=1,nc)
      write(36,105) (ncrare2(ii),ii=1,nc)

    call snp_count
       write(40,101) gho01,gao01
       write(41,105) (ncho01(ii),ii=1,nc)
       write(42,105) (ncao01(ii),ii=1,nc)

end do ! end of 1-t generation
!-----------------------------------
    do iii=1,nc
    do ii=1,2
    do jj=1,nl
      code=snpmaa(1,iii,ii,jj)
      inmaa(iii,ii,jj)=code
      ncmaa(jj,ii)=code
    end do
    end do
      write(50,501) ((ncmaa(j1,j2),j2=1,2),j1=1,nl)
      write(50,*)
    end do
!----------------------------------------------

write(21,*)
write(22,*)
write(23,*)
write(24,*)
write(25,*)
write(26,*)
write(27,*)
write(28,*)
write(30,*)
write(31,*)
write(32,*)
write(33,*)
write(34,*)
write(35,*)
write(36,*)
write(40,*)
write(41,*)
write(42,*)

end do ! end of 1-n replication
!---------------------------------------------------
close(21);close(22);close(23);close(24);close(25)
close(26);close(27);close(28);close(29)
close(30)
close(31);close(32);close(33);close(34);close(35);close(36)
close(40);close(41);close(42)
close(50)

101 format(2f10.4)
102 format(5f15.4)
103 format(2i10)
104 format(12i10)
105 format(19f10.4)
106 format(20f10.4)
107 format(100f10.4)
108 format(6f10.4)
501 format(2i5)
```

```
      end ! program of the main block

##snp_ncdiv.for
       subroutine snp_ncdiv


       implicit none

       common /resultaa/aa

       common /rncdiver01/ncae
       common/rncdiver02/ncao
       common/rncdiver03/nche
       common/rncdiver04/ncnp
       common/rncdiver05/ncpp
       common/rncdiver06/ncrare1
       common/rncdiver07/ncrare2


       integer,parameter::nc=19        !number of chromosomes
       integer,parameter::nl=1000    !number of marker

       real,dimension(nc,nl,2)::aa
       real,dimension(2)::freq_allele

       real,dimension(nc)::ncae,ncao,nche,ncnp,ncpp
       real,dimension(nc)::ncrare1,ncrare2

       integer ii,i,j

       integer ncnum
       integer temprare1,temprare2

       real temp ! frequency of snp
       real tempae,temphe,tempppp
       real lociae
       integer tempnp


   !----------- the start of calculation    ----------------

       do ii=1,nc
          ncnum=0
          tempae=0;temphe=0;tempppp=0;tempnp=0
          temprare1=0;temprare2=0

        do i=1,nl

          lociae=0

        do j=1,2
          freq_allele(j)=aa(ii,i,j)
          temp=freq_allele(j)

          lociae=lociae+temp*temp ! effective allelic rich

            if(temp>=0.001) then
               ncnum=ncnum+1             ! allelic number
            end if

          if(0.001<=temp.and.temp<0.02) then
               temprare1=temprare1+1 ! rare allele
```

```
              end if

            if(0.001<=temp.and.temp<0.05) then
                temprare2=temprare2+1
              end if
          end do ! end of a locus

                temphe=temphe+(1-lociae)
                tempae=tempae+1.0/lociae

                if (ncnum>1) then
                  tempnp=tempnp+1
                end if

              end do ! end of cycle snp loci in a given chromsome

              ncao(ii)=float(ncnum)/float(nl)    !ncnum is gonomic allelic number
              nche(ii)=temphe/float(nl)               !temphe is gonomic he
              ncae(ii)=tempae/float(nl)                !tempae is gonomic ae
              ncpp(ii)=float(tempnp)/float(nl) !tempp is gonomic pp
              ncnp(ii)=float(tempnp)                   !num_p is gonomic np
              ncrare1(ii)=float(temprare1)
              ncrare2(ii)=float(temprare2)
          end do



    end subroutine snp_ncdiv

##snp_output02.for
     subroutine snp_output

        implicit none



       common /popmaa/snpmaa

      integer,parameter::nc=19        !number of chromosomes
      integer,parameter::nl=1000      !number of marker
      integer,parameter::nmale=20 !number of male

      integer,dimension(nmale,nc,2,nl)::snpmaa


       integer code

       integer i,ii,iii
       integer j,jj,jjj

        do iii=1,nc
        do ii=1,nl
        do i=1,2
        do j=1,nmale
          code=snpmaa(j,iii,i,ii)
          snpmnl(iii,ii,i,j)=code
        end do
        end do
        end do
        end do

      do iii=1,nc
        do ii=1,nl
```

```
    do i=1,2
    do j=1,nmale
      code=snpmnl(iii,ii,i,j)
      ncmsnp(i,j)=code
    end do
    end do
       write(30,300) ((ncmsnp(jjj,jj),jj=1,nmale),jjj=1,2)
    end do

    end do

  close(30)

!#######################################################




  300 format(1x,20i4)
  305 format(1x,100i4)


  end subroutine snp_output
```

##snp_R02.for

```
  subroutine snp_R02

  ! this is reproduction subroutine, equal family random mating policy

  implicit none

    common /comseed/seed

    common /popmale/snpmale
    common /popfemale/snpfemale
    common /popmaa/snpmaa
    common /popfaa/snpfaa


    common/cross/crossover !connected with poisson block

    common /femalerandom/female_num
    common /malerandom/male_num ! connected with random_mating 2011

  integer,parameter::nc=19          !number of chromosomes
  integer,parameter::nl=1000        !number of marker
  integer,parameter::nmale=20       !number of male
  integer,parameter::nfemale=100 !number of female

  integer,parameter::matingratio=5

  integer,dimension(nmale,nc,2,nl)::snpmale,new_male
  integer,dimension(nfemale,nc,2,nl)::snpfemale,new_female

  integer,dimension(nmale,nc,2,nl)::snpmaa,new_maa
  integer,dimension(nfemale,nc,2,nl)::snpfaa,new_faa


  integer,dimension(nc,nl)::gmale,gfemale !gamete of individuals
  integer,dimension(nc,nl)::gmcod,gfcod

  integer,dimension(nl)::gg1,gg2,gtemp1,gtemp2
```

```
   integer,dimension(nl)::gcod1,gcod2
   integer,dimension(nl)::gctemp1,gctemp2

   ! temperal gametes based on chromsomes
   integer,dimension(nc)::crossover ! numbers of crossover per chromsome
   integer icross,num_cross !number or site of crossover
   integer kk ! counter for crossover

   integer,dimension(nc,2,nl)::snpm1
   integer,dimension(nc,2,nl)::snpf1
! genetic snp-code of the parent-offspring generations

   integer,dimension(nc,2,nl)::codm1
   integer,dimension(nc,2,nl)::codf1

   integer,dimension(nfemale)::female_num
   integer,dimension(nmale)::male_num


    integer,dimension(1):: seed
    real r

    integer i,ii,j,jj,jjj
    integer imale,ifemale
    integer k,num
    integer i1,i2

    external poisson_process
    external random_mating2011

    call random_seed(put=seed)

  ! *****************************************************
  ! to produce the next_genaration male population
  ! open(20,file='f:\dataresult\snpmgamete20.txt')
  ! open(21,file='f:\dataresult\snpmgamete21.txt')

  call random_mating2011

      do i=1,nmale ! choice of male and female by chance

     imale=male_num(i)

     call random_number(r)
      num=int(r*matingratio+1)
      i1=num+(i-1)*matingratio
      ifemale=female_num(i1)

        do j=1,nc
        do jj=1,2
        do jjj=1,nl
           snpm1(j,jj,jjj)=snpmale(imale,j,jj,jjj)
           snpf1(j,jj,jjj)=snpfemale(ifemale,j,jj,jjj)

           codm1(j,jj,jjj)=snpmaa(imale,j,jj,jjj)
           codf1(j,jj,jjj)=snpfaa(ifemale,j,jj,jjj)

        end do
        end do
        end do

! write(20,201) imale
! write(21,201) imale
```

```
! &&&&&&&&&&&& producation of male gamete &&&&&&&&&&&&&&&&

      do j=1,nc ! producation of male gamete

           do jjj=1,nl
              gg1(jjj)=snpm1(j,1,jjj)
              gg2(jjj)=snpm1(j,2,jjj)

              gcod1(jjj)=codm1(j,1,jjj)
              gcod2(jjj)=codm1(j,2,jjj)

           end do

!11 -------- crossover ------------------------
!      write(20,200) (gg1(ii),ii=1,nl)
!      write(20,200) (gg2(ii),ii=1,nl)

           call poisson_process
           num_cross=crossover(j)

           if(num_cross>=1) then
             do kk=1,num_cross ! kk cycle
                do jjj=1,nl
                   gtemp1(jjj)=gg1(jjj)
                   gtemp2(jjj)=gg2(jjj)
                   gctemp1(jjj)=gcod1(jjj)
                   gctemp2(jjj)=gcod2(jjj)
                end do
                call random_number(r)
                 icross=int(r*nl)
                do jjj=1,nl
                  if(jjj<=icross) then
                   gg1(jjj)=gtemp1(jjj)
                   gg2(jjj)=gtemp2(jjj)

                   gcod1(jjj)=gctemp1(jjj)
                   gcod2(jjj)=gctemp2(jjj)
                  else
                   gg1(jjj)=gtemp2(jjj)
                   gg2(jjj)=gtemp1(jjj)

                   gcod1(jjj)=gctemp2(jjj)
                   gcod2(jjj)=gctemp1(jjj)
                  end if
               end do
             end do ! end of cycle kk
           end if
! write(21,*) num_cross
! write(21,200) (gg1(ii),ii=1,nl)
! write(21,200) (gg2(ii),ii=1,nl)
!+++++++++++++++++++++++++++++++++++++++++++++++++++++++++++++
!11 -- the end of function of crossover for male gametes

         call random_number(r)
            num=int(r*1000)
            k=mod(num,2)

            if(k==0)then
              do jjj=1,nl
                gmale(j,jjj)=gg1(jjj)
                gmcod(j,jjj)=gcod1(jjj)
              end do
```

```
            else

              do jjj=1,nl
                gmale(j,jjj)=gg2(jjj)
                gmcod(j,jjj)=gcod2(jjj)
              end do
            end if

     end do ! end cycle (nc)
! &&&&&&&&&&&& producation of male gamete &&&&&&&&&&&&&&&&---

   do j=1,nc ! producation of female gamete

        do jjj=1,nl
            gg1(jjj)=snpf1(j,1,jjj)
            gg2(jjj)=snpf1(j,2,jjj)

            gcod1(jjj)=codf1(j,1,jjj)
            gcod2(jjj)=codf1(j,2,jjj)
        end do
        call poisson_process
           num_cross=crossover(j)
           if(num_cross>=1) then

           do kk=1,num_cross
               do jjj=1,nl

                   gtemp1(jjj)=gg1(jjj)
                   gtemp2(jjj)=gg2(jjj)

                   gctemp1(jjj)=gcod1(jjj)
                   gctemp2(jjj)=gcod2(jjj)

               end do
               call random_number(r)
                  icross=int(r*nl)
                 do jjj=1,nl
                     if(jjj<icross) then
                       gg1(jjj)=gtemp1(jjj)
                       gg2(jjj)=gtemp2(jjj)

                       gcod1(jjj)=gctemp1(jjj)
                       gcod2(jjj)=gctemp2(jjj)

                     else
                       gg1(jjj)=gtemp2(jjj)
                       gg2(jjj)=gtemp1(jjj)

                       gcod1(jjj)=gctemp2(jjj)
                       gcod2(jjj)=gctemp1(jjj)

                     end if
                 end do
            end do ! end of crossover
            end if

              call random_number(r)
                num=int(r*1000)
                k=mod(num,2)
              if(k==0)then
                do jjj=1,nl
                  gfemale(j,jjj)=gg1(jjj)
                  gfcod(j,jjj)=gcod1(jjj)
```

```
                 end do
               else
                 do jjj=1,nl
                   gfemale(j,jjj)=gg2(jjj)
                   gfcod(j,jjj)=gcod2(jjj)

                 end do
               end if
         end do ! end of nc for female gametes
!++++++++++++++++++++++++++++++++++++++++++++++++++++++++
!producation of next generation of malepop
            do j=1,nc
            do jjj=1,nl
               new_male(i,j,1,jjj)=gfemale(j,jjj)
               new_male(i,j,2,jjj)=gmale(j,jjj)

               new_maa(i,j,1,jjj)=gfcod(j,jjj)
               new_maa(i,j,2,jjj)=gmcod(j,jjj)
            end do
            end do

        end do ! the end to produce the next_genaration male population

! close(20)
! ******************************************************************
! to produce the next_genaration female population

   do i=1,nmale      !choice of male and female by chance

          imale=male_num(i)

          do j=1,nc
          do jj=1,2
          do jjj=1,nl
             snpm1(j,jj,jjj)=snpmale(imale,j,jj,jjj)

             codm1(j,jj,jjj)=snpmaa(imale,j,jj,jjj)
          end do
          end do
          end do

       do ii=1,matingratio
             i2=ii+(i-1)*matingratio
             ifemale=female_num(i2)
          do j=1,nc
          do jj=1,2
          do jjj=1,nl
             snpf1(j,jj,jjj)=snpfemale(ifemale,j,jj,jjj)

             codf1(j,jj,jjj)=snpfaa(ifemale,j,jj,jjj)
          end do
          end do
          end do

! &&&&&&&&&&&& producation of male gamete &&&&&&&&&&&&&&&&---
          do j=1,nc ! producation of male gamete
             do jjj=1,nl
                gg1(jjj)=snpm1(j,1,jjj)
                gg2(jjj)=snpm1(j,2,jjj)

                gcod1(jjj)=codm1(j,1,jjj)
                gcod2(jjj)=codm1(j,2,jjj)
```

```
        end do
!11 -------- crossover -----------------------
        call poisson_process
        num_cross=crossover(j)
        if(num_cross>=1) then
          do kk=1,num_cross ! kk cycle
            do jjj=1,nl
              gtemp1(jjj)=gg1(jjj)
              gtemp2(jjj)=gg2(jjj)

              gctemp1(jjj)=gcod1(jjj)
              gctemp2(jjj)=gcod2(jjj)

            end do
            call random_number(r)
             icross=int(r*nl)
            do jjj=1,nl
              if(jjj<=icross) then
               gg1(jjj)=gtemp1(jjj)
               gg2(jjj)=gtemp2(jjj)

               gcod1(jjj)=gctemp1(jjj)
               gcod2(jjj)=gctemp2(jjj)
              else
               gg1(jjj)=gtemp2(jjj)
               gg2(jjj)=gtemp1(jjj)

               gcod1(jjj)=gctemp2(jjj)
               gcod2(jjj)=gctemp1(jjj)

              end if
             end do
          end do ! end of cycle kk
        end if

      !11 -- the end of function of crossover for male gametes
      call random_number(r)
         num=int(r*1000)
         k=mod(num,2)

         if(k==0)then
           do jjj=1,nl
             gmale(j,jjj)=gg1(jjj)
             gmcod(j,jjj)=gcod1(jjj)
           end do
         else
           do jjj=1,nl
             gmale(j,jjj)=gg2(jjj)
             gmcod(j,jjj)=gcod2(jjj)

           end do
         end if

    end do ! end cycle (nc)
  ! &&&&&&&&&&&& producation of male gamete &&&&&&&&&&&&&&&&---

   do j=1,nc ! producation of female gamete
      do jjj=1,nl
         gg1(jjj)=snpf1(j,1,jjj)
         gg2(jjj)=snpf1(j,2,jjj)

         gcod1(jjj)=codf1(j,1,jjj)
         gcod2(jjj)=codf1(j,2,jjj)
```

```
          end do
          call poisson_process
             num_cross=crossover(j)
             if(num_cross>=1) then
             do kk=1,num_cross
                  do jjj=1,nl
                       gtemp1(jjj)=gg1(jjj)
                       gtemp2(jjj)=gg2(jjj)

                       gctemp1(jjj)=gcod1(jjj)
                       gctemp2(jjj)=gcod2(jjj)

                  end do
                  call random_number(r)
                     icross=int(r*nl)
                    do jjj=1,nl
                         if(jjj<icross) then
                           gg1(jjj)=gtemp1(jjj)
                           gg2(jjj)=gtemp2(jjj)

                           gcod1(jjj)=gctemp1(jjj)
                           gcod2(jjj)=gctemp2(jjj)
                         else
                           gg1(jjj)=gtemp2(jjj)
                           gg2(jjj)=gtemp1(jjj)

                           gcod1(jjj)=gctemp2(jjj)
                           gcod2(jjj)=gctemp1(jjj)

                         end if
                    end do
              end do ! end of crossover
              end if

                call random_number(r)
                  num=int(r*1000)
                  k=mod(num,2)
                if(k==0)then
                  do jjj=1,nl
                    gfemale(j,jjj)=gg1(jjj)

                    gfcod(j,jjj)=gcod1(jjj)
                  end do
                else
                  do jjj=1,nl
                    gfemale(j,jjj)=gg2(jjj)
                    gfcod(j,jjj)=gcod2(jjj)

                  end do
                end if
      end do ! end of nc for female gametes

! producation of next generation of femalepop
           do j=1,nc
           do jjj=1,nl
              new_female(i2,j,1,jjj)=gfemale(j,jjj)
              new_female(i2,j,2,jjj)=gmale(j,jjj)

              new_faa(i2,j,1,jjj)=gfcod(j,jjj)
              new_faa(i2,j,2,jjj)=gmcod(j,jjj)

           end do
```

```
          end do
       end do ! the end of mating_ratio
       end do ! the end to produce the next_genaration male population
       call random_seed(get=seed)

       snpmale=new_male
       snpfemale=new_female

       snpmaa=new_maa
       snpfaa=new_faa

! open(22,file='f:\dataresult\snpmapmale.txt')
!
!
!        do i=1,nmale
!
!           write(22,200) i
!
   !     do j=1,nc
!           do jj=1,2
   !              do jjj=1,nl
!                snpm1(j,jj,jjj)=snpmale(i,j,jj,jjj)
!                 gmale(jj,jjj)=snpmale(i,j,jj,jjj)
!            end do
!            end do
!                write(22,200) ((gmale(j,jjj),jjj=1,nl),j=1,2)
!           end do
!           end do
!   close(22)


      200 format(1x,100i1)
      201 format(1x,i3)

       end subroutine snp_R02
```
